# Diurnal variation in expired breath volatiles in malaria-infected and healthy volunteers

**DOI:** 10.1088/1752-7163/aadbbb

**Published:** 2018-09-19

**Authors:** Amalia Z Berna, James S McCarthy, X Rosalind Wang, Michelle Michie, Florence G Bravo, Julie Cassells, Stephen C Trowell

**Affiliations:** 1 Department of Pediatrics, Washington University School of Medicine, St. Louis, MO63110, United States of America; 2 CSIRO Health and Biosecurity, Clunies-Ross Street, Acton ACT 2601, Australia; 3 QIMR Berghofer Medical Research Institute. University of Queensland and Dept. of Infectious Diseases Royal Brisbane and Womens Hospital, 300 Herston Road, Herston QLD4006, Australia; 4 CSIRO Data61, Corner of Vimiera and Pembroke Roads, Marsfield NSW2122, Australia

**Keywords:** thioethers, terpenes, biomarker, breath, malaria, *Plasmodium falciparum*, *Plasmodium vivax*

## Abstract

We previously showed that thioether levels in the exhaled breath volatiles of volunteers undergoing controlled human malaria infection (CHMI) with *P. falciparum* increase as infection progresses. In this study, we show that thioethers have diurnal cyclical increasing patterns and their levels are significantly higher in *P. falciparum* CHMI volunteers compared to those of healthy volunteers. The synchronized cycle and elevation of thioethers were not present in *P. vivax*-infection, therefore it is likely that the thioethers are associated with unique factors in the pathology of *P. falciparum*. Moreover, we found that time-of-day of breath collection is important to accurately predict (98%) *P. falciparum*-infection. Critically, this was achieved when the disease was asymptomatic and parasitemia was below the level detectable by microscopy. Although these findings are encouraging, they show limitations because of the limited and logistically difficult diagnostic window and its utility to *P. falciparum* malaria only. We looked for new biomarkers in the breath of *P. vivax* CHMI volunteers and found that a set of terpenes increase significantly over the course of the malaria infection. The accuracy of predicting *P. vivax* using breath terpenes was up to 91%. Moreover, some of the terpenes were also found in the breath of *P. falciparum* CHMI volunteers (accuracy up to 93.5%). The results suggest that terpenes might represent better biomarkers than thioethers to predict malaria as they were not subject to malaria pathogens diurnal changes.

## Introduction

Efforts to control malaria require accurate and reliable diagnostic methods. Although historically microscopy has been the most common way to diagnose malaria, by 2014, rapid diagnostic tests (RDTs) accounted for 71% of all diagnostic testing for suspected malaria [1]. RDTs detect a number of protein biomarkers that the malaria parasite releases into the bloodstream, including histidine rich protein 2 (HRP2) and lactate dehydrogenase. Unfortunately, RDTs are not sufficiently sensitive to detect *Plasmodium* parasites at low levels of parasitemia, when infected individuals can act as reservoirs for ongoing malaria transmission [2]. As well, the recent emergence of *P. falciparum* parasites with partial or complete deletion of the *pfhrp2* gene has led to the worrying occurrence of false-negative RDT results with potentially clinically significant adverse health effects [3–7]. For these reasons, new diagnostic modalities are therefore required.

Recently, we identified volatile biomarkers of *Plasmodium falciparum* infection in the breath of volunteers undergoing a controlled human malaria infection (CHMI) using the induced blood stage infection model (IBSM)[8]. The levels of four sulfur-compounds in the volunteers’ breath consistently roseover the course of the infection and dropped following treatment. These changes in thioether levels were detected at very low parasitemia, before either blood films became positive or the volunteers became symptomatic. Furthermore, we noted cyclical increases and decreases in thioether levels imposed on the general trend. However, the low frequency of sampling prevented precise determination of the period of the cycle.

The cyclical nature of thioether production was a novel observation consistent with general findings about diurnal variations in levels of volatile organic compounds (VOCs) in exhaled breath. Investigation and understanding of temporal patterns in breath volatiles are important to develop novel diagnostic tools and to maximize the efficacy and safety of new or existing drugs [9]. Earlier studies have shown that the levels of the abundant endogenous breath VOCs, acetone and isoprene, increase overnight and decline during the day [10]. Sinues *et al* [11] monitored diurnal changes in exhaled breath volatiles (EBV) and found that, for example, a compound characterized by an ion with the mass to charge ratio (*m/z*) of 211 increased steadily during the morning, reached a maximum shortly after lunch, and declined during the late afternoon. In contrast, levels of another compound with *m/z* = 199 declined progressively from morning to afternoon. More recently, Martinez-Lozano *et al* [12] studied variations in levels of breath volatiles in three healthy individuals during total sleep deprivation, using secondary electrospray ionization mass spectrometry. They found 111 features in the breath of individuals, of which 36%–39% showed significant diurnal variation in at least one individual. Although the study did not unambiguously identify compounds, it demonstrated sizable time-of-day variations in breath metabolite levels and highlighted the possible significance of time of day variation when assessing EBV as biomarkers of disease.

In this study, we investigated the periodicity of changes in thioether levels in exhaled breath of volunteers undergoing CHMI with *Plasmodium falciparum* and *P. vivax* and compared the results with breath volatiles levels in uninfected volunteers. We also searched for additional novel breath biomarkers in the exhaled breath of volunteers undergoing CHMI with either *Plasmodium falciparum* or *P. vivax* species, using an untargeted approach. In addition to the significance of the discovery of novel breath biomarkers for malaria, understanding diurnal cycles in exhaled breath is important for designing optimal breath sampling strategies for breath-based diagnosis.

## Methods

### CHMI trials

*P. falciparum* and *P. vivax* CHMI trials were conducted as previously described [13, 14]. Separate studies were undertaken to test the effectiveness of antimalarial agents against *P. falciparum* and *P. vivax*, the results of which (including parasitaemia after treatment) will be reported elsewhere. The antimalarial drugs used were MMV 390048 for *P. falciparum* trial and OZ439 for *P. vivax* trial. Written informed consent to participate in the breath analysis arm of the study, in addition to the drug trial, was received from participants in the study. Trial were registered in ClinicalTrials.gov with identifier number: NCT02783833 for the *P. falciparum* trial and NCT02573857 for the *P. vivax* trial. Descriptions of the clinical trials, ethics approvals and polymerase chain reaction (PCR) method for quantification of *P. falciparum* and *P. vivax* parasitaemia [13, 15] are presented in supplementary material S2.1 is available online at stacks.iop.org/JBR/12/046014/mmedia.

### Exhaled breath collection

CHMI and healthy control volunteers breathed through a cardboard mouthpiece connected to a chamber. The chamber was then attached using tubing to a 3-L SamplePro FlexFilm sample bag (SKC Inc, Pennsylvania). The volunteers were asked to take a few deep breaths and on the final breath in to undertake a small exhalation using a short ‘ha’ expiration, pause, and during the pause, put the cardboard tube between the lips and continue the exhalation as far as they could, comfortably. This process was designed to selectively collect the later portion of the exhaled enriched alveolar air. Neither a nose clip nor VOC filter were used. Breath from the bags was transferred to a sorbent tube as previously described [8]. Breath samples were collected from each volunteer for up to 10 days for the *P. falciparum* trial and for up to 15 days for the trial *P. vivax*. Breath collection time points are detailed in supplementary table 1. For healthy controls, we recruited eight participants. Each participant provided: four breath samples containing at least 1 L of breath daily at the following times: in the morning (around 7:00), 11:00, 15:00 and 19:00. Samples were collected from all participants for three consecutive days. Longer period of breath collection per participant was not possible due to logistical limitations at the time the experiments were carried out total number of samples collected was 96 = 8 volunteers × 3 days × 4 time points. To account for day effect and allow better comparison with infected samples that were collected over a longer period of time (10 and 15 days), the healthy control samples were collected over a period of 3 weeks, i.e. three volunteers in Week 1 and Week 2 and two volunteers in Week 3 (see supplementary tables 1(B) and 1(C) for details).

**Table 1:** Retention times (Rt) and mass to chargeratios (*m/z*) for the informative features identified in GC-QTOF-MS analysis of breath samples from volunteers infected with *P. vivax*.

Rt(min)	Mass to charge ratio (*m/z*)	Compound identification
14.52–14.75	91, 136, 118, 89, 120, 134, 63	α-terpinene
14.80	89, 104, 134, 120	m-cymene
14.96	153	Unknown 1
15.01	92	Limonene
15.04	147	Unknown 2
17.10	132, 92, 76	Terpinolene
28.98	167	Unknown 3

### GC-MS analysis of breath samples

Breath samples were analyzed using quadrupole time of flight GC-MS (7890B series, Agilent Technologies). Volatiles were thermally desorbed from the sorbent tubes (Unity2, Markes International) and chromatograms were analyzed using the Mass Hunter Qualitative analysis software. Deconvolution was used to identify compounds. Ion extraction was used to calculate the area under peak for each thioether. In addition, chemical standards were used to confirm the identities of compounds. Detailed description of gas chromatography mass spectrometry and subsequent analysis of the captured breath samples can be found in supplementary material S2.2 and S2.3.

### Statistic alanalysis

#### Fold change analysis

We used fold change (FC) as a measure to describe how much the mean level of a breath VOC changes between healthy and malaria-infected individuals in samples collected at similar time points in both data sets. FC was also used to describe changes in VOC levels between an original and subsequent measurement within the same data set. We presented the fold change as a ratio between the two levels being calculated. To test the null hypothesis that FC = 1, which implies that the two means being compared are equal, the Mann–Whitney test (nonparametric test) was used. We define a statistically significant difference in means if the p-value is less than 0.05.

#### Multicomparison test

In this work, we applied t-tests (with Bonferroni correction; 95% confidence interval) to the thioether levels of the healthy controls in order to find if there were changes between paired time of day samples. The Bonferroni correction is used to reduce the chances of obtaining false-positive results (type I errors) when multiple pairwise tests are performed on a single set of data. T-tests were performed using GraphPad Software(version 7.03).

#### Machine learning classification of thioether levels in CHMI *P. falciparum* data and healthy controls

Machine learning algorithms are potentially able to identify patterns in data that are not obvious to human inspection, due to the size and complexity of the data. For example, machine learning has been shown to perform well in predicting the recurrence of lung cancer or patient prognosis [16]. However rigorous efforts are necessary to identify suitable data features and to avoid overfitting when developing machine learning-based predictors[17].

In this work, support vector machine (SVM) [18] and k-nearest neighbor (k-NN)[19] machine learning algorithms were used to test if the EBV data can support classification, i.e. is it possible to correctly classify infected and non-infected volunteers? We present results using linear SVM_L_ and kNN (*k* = 3) algorithms. Other parameters, kNN *k* = 5 (as the total number of samples were 7) and SVM_R_ (radial), gave similar results to *k* = 3 and can be seen in supplementary data. SVM was performed using the open source software LIBSVM [20]; KNN and feature selection were performed using in-house software written in MATLAB.

To perform classifications, only breath samples collected at 19:00 from *P. falciparum* volunteers were used. This was done because samples collected at 19:00 had elevated levels of the thioethers in *P. falciparum* CHMI. Twenty three breath samples from heathy controls were available to construct classifications (8 volunteers × 3 days at 19:00; with one missed sample). Classification was done using five random subsets of data each containing seven (out of 23) healthy samples. This was done to ensure the resultant data is balanced (i.e. similar number of samples in each class). We report here the mean results for the five sets. Full results are shown in supplementary data.

Leave-one-out cross-validation was used to train and test classifiers. The full data set was partitioned into *N* pairs of a training set (of *N* − 1 data samples) and a test set (of the remaining 1 data sample). This process was repeated *N* times to cover each data point in the data set, once only.

#### Hypothesis test for classification

A statistically significant classification result is one where we can reject the null hypothesis that the biomarkers present no information about infection status. The null hypothesis in classification is where the classifier is operating at chance. If a classifier is operating at random, then we expect the accuracy to be on average *r/N*, where *r* is the number of samples in the class with the highest number of samples in the data set [21]. The *p*-value under the null hypothesis for *y* instance of correctly labeled samples is *P(X ≥ y)*, where *X* is a random variable with binomial distribution with *N* samples, and probability of success *r/N*. We define a statistically significant classification accuracy if the *p*-value is less than 0.05.

The accuracy of the test is defined as the ability to differentiate the infected samples and healthy controls correctly using the thioethers. Sensitivity and specificity of the test are also calculated here and they refer to the ability of the test to determine the infected samples correctly (sensitivity) and healthy controls correctly (specificity).

#### Feature selection for untargeted search of biomarkers in *P. vivax* trials

We took an automated, machine learning approach to search for volatiles associated with the pre-treatment course of infection in the *P. vivax* cohort. First, the GC-MS data from the *P. vivax* CHMI trial was preprocessed in the following steps: (1) normalization, (2) baseline correction, (3) peak detection, and (4) peak alignment. A full description of the method can be found in supplementary data S2.5. After this process was completed, we calculated the mutual information (MI)[22, 23] between each ‘feature’ (unique combinations of a specific value of mass to charge ratio (*m/z*) and the GC retention time (RT) with the time course (i.e.stage of the infection).

#### Machine learning classification of infected cases and healthy controls using biomarkers found through untargeted search

In the same way as the thioethers, we used SVM and kNN machine learning algorithms to test if the data can support a classification. We present results using linear SVM_L_ and kNN algorithms value of *k* = 3 for both *P. falciparum* and *P. vivax* CHMI. Other parameters, kNN *k* > 3 and SVM_R_ (radial), gave similar performance and can be seen in supplementary data. To perform classifications, we used all breath samples collected from Day 6 after infection until just before the antimalarial drug was given, this resulted in seven time points for *P. falciparum* and six time points for the *P. vivax* data. A total 48 data points was available for classification in each trial.

A total number of 95 breath samples were available from healthy controls to construct the classifications (8 volunteers × 3 days × 4 time points; 1 lost sample). We randomly sample the full dataset fivetimes and each data set contains 48 samples. This was done to ensure datasets with balanced number of samples in both infected and control classes and make sure that the results are not due to random sampling in the subset. We then performed classification on each of the five sub-sets and the average results are discussed hereafter.

We used the same cross-validation procedures and test for statistical significance on new biomarkers found through untargeted search (terpenes) as for the thioether data.

We also trained the classifiers using the terpene data (48 Pf versus 48 controls) and used it to classify the *P. vivax* and healthy data (48 samples for *P. vivax* and the remaining 47 healthy data not included in the training data set). Conversely, we trained the classification on *P. vivax* and healthy data and used these to classify the *P. falciparum* and healthy data. This was done to guard against overfitting of data. Classifications were performed for those compounds whose identity was confirmed by standards.

### Chemicals

Allyl methyl sulfide (HMDB31653) was purchased from Sigma-Aldrich (Belgium) and 1-methylthio-propane (HMDB61871) from ABCR GmbH & Co (Karlsruhe,Germany). (E)-1-methylthio-1-propene (HMDB59843) and (Z)-1-methylthio-1-propene (HMDB61876) were synthesized by Advanced Molecular Technologies Ptyltd (Melbourne, Australia).

Synthetic standards of the following terpenes: α-terpinene (HMDB36995), terpinolene (HMDB36994), m-cymene (HMDB37051) were purchased from Sigma-Aldrich (Belgium). Limonene (HMDB03375) was purchased from Honeywell Fluka(USA).

## Results

### Periodicity of potentially diagnostic low levels VOCs was found in the breath of P. falciparum volunteers

In an IBSM study undertaken in 2016 with *P. falciparum*, breath samples were collected from seven volunteers. To better define the periodicity of changes in the levels of thioethers compared to our previous studies [8], we increased the frequency of breath collection to up to four times per day at approximately four hour intervals, during waking hours. Details of breath collection time points can be found in supplementary table 1.

Volunteers with *P. falciparum* CHMI became PCR positive on Day 4 (95.8 h) after inoculation, and reached the treatment threshold of >1000 parasites ml^−1^ on the morning of Day 8 (190.7 h after inoculation), when the volunteers were admitted to the clinic and designated treatment was commenced.

**Figure 1:**
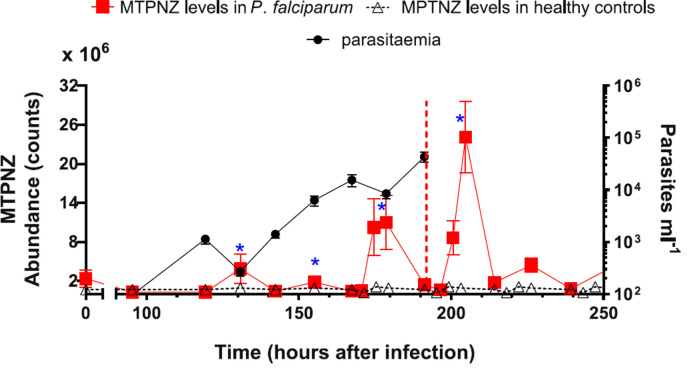
Time course of mean levels of MTPNZ for *P. falciparum* IBSM trials (N = 7), healthy controls (N = 8) and parasitaemia. *P. falciparum* infection was initiated immediately after the time zero breath sample. The red vertical dotted line represents the time when antimalarial treatment was administered in the *P. falciparum* trial. The healthy control data shows the mean levels of the compound found at that time of day, derived from three consecutive days of measurements. The diurnal cycle is repeated over the duration of the IBSM trial, and the collection time matched to collections from the *P. falciparum*-infected subjects. Figure shows mean (SEM). SEM is used as a measure of precision for the estimated mean. Blue asterisks denote sampling times between 19:00 and 21:00.

At Day 4 (95.8 h) after infection, the first measurement of the day (between 7:00 and 9:00) showed that the concentrations of three thioethers: 1-methylthiopropane (MTP), E-1-methylthio-1-propene (MTPNE) and Z-1-Methylthio-1-propene (MTPNZ), was approximately ten-fold lower compared with levels at Day 1 (baseline). This difference was statistically significant at p= 0.003. There after, we observed synchronized cyclical changes in the levels of MTP, MTPNE and MTPNZ with a period of approximately 24 h. Compared to the lowest MTPNZ level (95.8 h after infection), the changes in MTPNZ concentration reached a significant (p = 0.001) 87-fold increase (95% CI: 35.15–454.16) about the time the infection became symptomatic (figure 1). There was a similar overall increase in the amplitudes of the variations in concentrations of MTPNE and MTP (supplementary figure 1).

On Day 8 (190.7 h), after volunteers were treated with an experimental antimalarial that contained no sulfur atoms, two different patterns were observed in the levels of thioethers: (1) one cycle of increase and decrease with a maximum ∼14 h after drug administration for MTP and MTPNE, followed by a steady decrease in levels (supplementary figure 1); (2) two cycles of increase and decrease with maxima reached at ∼14 and at ∼38 h after drug administration, was observed for MTPNZ (figure 1). Cycle periods were between approximately 19 and 24 h. The pattern of changes for each of the thioethers, in most of the volunteers, was similar (supplementary figure 2).

To determine whether the synchronized cyclical change in the levels of thioethers in EBV is unique to individuals infected with *P. falciparum*, breath samples of healthy individuals were collected and analyzed.

Thioether levels were measured in eight healthy volunteers four times a day (at approximately 7:00, 11:00, 15:00 and 19:00). The results showed that the concentrations of the thioethers rose and fell by a factor of four over the course of the day. The lowest level of thioethers in the breath of volunteers was at 11:00 (supplementary figure 3). A multicomparison test (Bonferroni; 95% confidence interval) revealed that, for MTPNZ, measurements at 11: 00 had significantly lower mean compared to measurements at 15:00 (*p* = 0.0028) and 19:00 (*p* = 0.028). For MTP and MTPNE there were significant differences (*p* < 0.05) between the ‘morning’ (7:00) and ‘before lunch’ (11:00) measurements. The concentration of AMS did not change significantly over the course of the day. Full results of the comparisons can be found in supplementary table 2.

In order to compare *P. falciparum* data with healthy controls, we calculated mean levels of the compound found for all 8 healthy control samples over the three days of analysis, this means *n* = 24 samples (8 volunteers × 3 days) at each time point. The diurnal cycle is repeated over the duration of the IBSM trial, and the collection time matched to collections from the *P. falciparum*-infected subjects (figure 1).

Thioether concentrations in the EBV of healthy individuals showed much lower diurnal variation than in *P. falciparum*-infected volunteers. The peak magnitudes of volatiles did not approach those seen at comparable time points of the *P. falciparum* trials (figure 1 and supplementary figure 1). For example, for MTPNZ, there is a 27-fold increase (95% CI: 11.83–63.45) in the means of the MTPNZ concentration in IBSM volunteers, compared to healthy controls. The difference is statistically significant at *p* < 0.001. Fold change and significance for all other thioethers is shown in supplementary table 3. This result supports the hypothesis that both the cycling and elevation in concentration of thioethers in EBV is specific to infection with *P. falciparum*.

We also investigated whether there was a relationship between thioether levels and parasitaemia levels. The higher frequency of sampling in this study compared to our previous study revealed that the cyclical changes in the levels of thioether have a period of 24 h. Parasitaemia cycles every 48 h. Moreover, parasitaemia changes are out of phase with the thioether levels. We found no direct relationship between the levels of the thioethers and parasitaemia. In the future, we aim to build machine learning regression models that can predict this intricate changes of both parasitaemia and breath volatiles.

### High predictive value of thioethers in EBV

**Figure 2:**
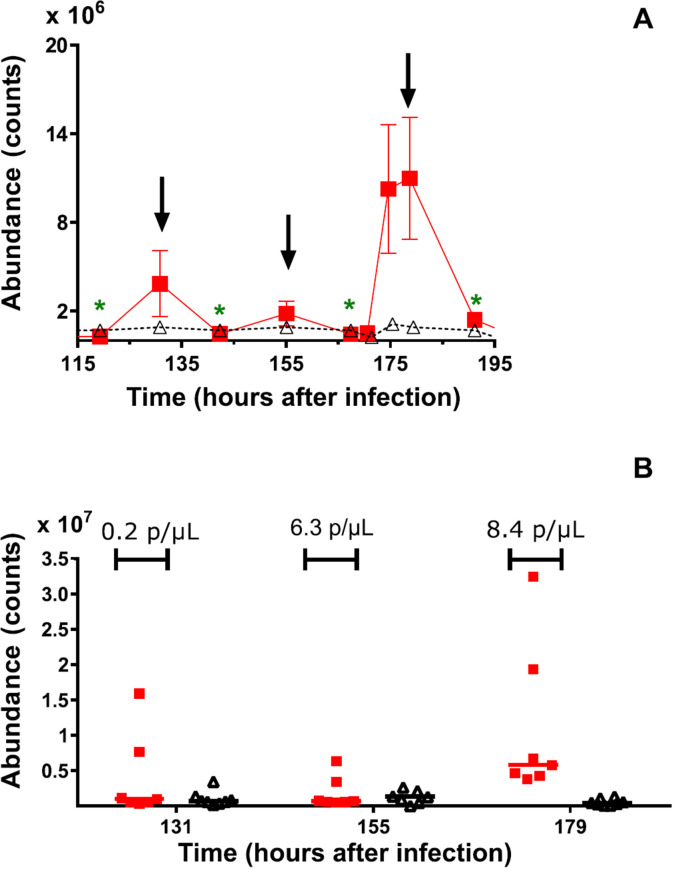
(A) Extract of time course of mean levels of (Z)-1-methylthio-1-propene for *P. falciparum* CHMI trials (N = 7) (∎) and for healthy controls (N = 8) (Δ). The healthy control data shows the mean levels of the compound at the specified time of day and were derived from three consecutive days of measurement. The diurnal cycle is repeated over the duration of the CHMI trial and the collection time matched to CHMI collections. Figure shows mean(sem). Arrows indicate the peak of the compound which corresponds to 12 h after the first sample of the day is collected (i.e. between 19:00 and 21:00). Green asterisks indicate sampling times between 7:00 and 8:00. (B) Comparisons of mean (Z)-1-methylthio-1-propene levels for *P. falciparum* (N = 7) CHMI trial (∎) and healthy controls (N = 8) (Δ) for samples collected between 19:00 and 21:00. For the CHMI trial, breath samples were collected at 131 h (Day 5), 155 h (Day 6) and 179 h (Day 7) after infection. Values above red symbols represent the average parasitaemia (parasites μl-1) at each breath collection time point.

Although thioether levels in the *P. falciparum* volunteers are, on average, elevated they drop to levels close to those of healthy volunteer severy 24 h (figure2(A)). If, however, comparisons are made 12 h after the first sample of the day is collected (i.e. between 19:00 and 21:00), at the peak of the thioether evolution, there is an up to 27-fold increase (95% CI: 11.83–63.45) in the means of the MTPNZ in *P. falciparum*-infected volunteers compared to healthy controls. Similar differences were observed for MTP and MTPNE (supplementary figure 1).

Based on this observation, we tested the hypothesis that there is significant difference between malaria-infected groups and healthy controls using one or more of the target thioethers, in samples collected between 19:00 and 21:00. The quality of this test was limited by the small size of the data set(*N* = 7).

The robustness of the target thioethers to discriminate between malaria-infected groups and controls, was evaluated by two standard classification algorithms from the field of machine learning: a SVM and kNN (described in supplementary data S2.5). The classification was considered statistically significant when the accuracy was greater than 71.4% (10/14 samples), i.e. 95% confidence interval level.

For the infected samples, we used those collected at night (i.e. 20:00 ± 1 h) for Day 5 (131 h), Day 6 (155 h) and Day 7 (179 h) after infection. For healthy samples, we generated data sets from randomly selected samples from the healthy control subjects at similar time points to the malaria-infected participants. This was done because there were a larger number of sampling time points available for healthy controls *N* = 24 (8 volunteers, repeated over 3 days) than for CHMI participants (*N* = 7) at each collection time. Moreover, the healthy sample set contains multiple breath samples from the same person, which was not the case in infected samples and could potentially bias the result in a leave-one-out cross-validation. Details of the random selection can be found in methods section.

At Day 5 (131 h) of infection, when the mean parasitaemia was 0.2(±0.05) parasites μl^−1^, none of the thioethers yielded classification accuracies above the significance level value (i.e. 71.43% at *p* = 0.05), meaning that there isnosignificant difference between malaria-infected groups and healthy controls at this level of parasitaemia (figure 2(B) and supplementary figure 4 and supplementary table 3(A)). At Day 6 (155 h after infection), when mean parasitaemia was 6.3(±1.3) parasites μl^−1^, two out of the eight tested classifications showed significant differences between the two groups. These two were AMS with classification accuracy of 75% and MTPNE with 80%, accuracy. AMS and MTPNE had specificities of 80% and 88.6% respectively and both compounds had sensitivities of 71.4% (supplementary tables 4(A) and (B)).

At Day 7 (179 h) of infection, when the subjects were still asymptomatic and parasitaemia below the level detectable by microscopy (mean parasitaemia of 8.4(±1.6) parasites μl^−1^) analysis of MTP, MPTNE and MPTNZ levels resulted in classification accuracies between 91% and 98%, which are consistent with the data shown in figure 2(B) and supplementary figure 4. AMS seems to have no predictive value (supplementary table 4(A)). MTP and MPTNZ achieved a diagnostic sensitivity of 100%. The sensitivity using MTPNE was 82.9%. The best specificity was achieved with MPTNE (100%) while MTP and MPTNZ showed 97.1% and 88.6% sensitivity, respectively. Together the compounds performed well in measuring true positive and true negative samples when parasitaemia was 8.4 parasites μl^−1^.

### No thioether periodicity was observed in P. vivax trials

We also investigated whether cyclical increases in thioether levels are observed in the volunteers with an experimental *P. vivax* infection.

**Figure 3:**
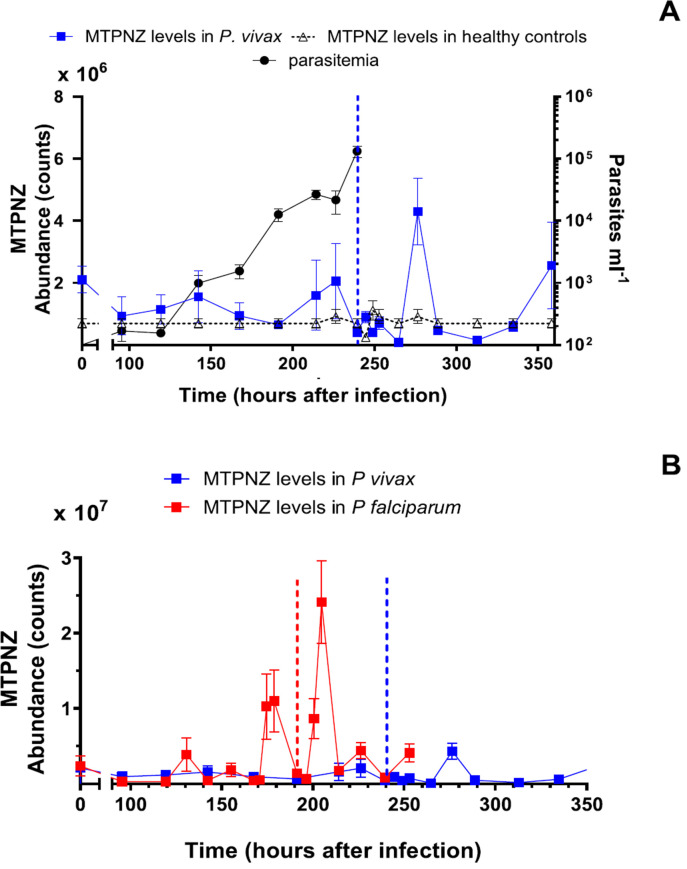
(A) Time course of levels of MTPNZ in breath of participants undergoing CHMI with *P. vivax* (*N* = 8), healthy controls (*N* = 8) and parasitaemia levels. Blue vertical dotted line represents the time when experimental antimalarial was administered. The healthy control data shows the mean levels of the compound found at the specified times of day, derived from three consecutive days of measurements. The diurnal cycle is repeated over the duration of the IBSM trial, and the collection time was matched to collections from the *P. vivax*-infected subjects. Figure shows mean(sem). (B) Comparisons of mean of MTPNZ thioether levels for *P. falciparum* (*N* = 7) and *P. vivax* (*N* = 8) CHMI trials. Both infections were initiated immediately after the baseline breath sample, taken at time zero. Red (*P. falciparum*) and blue (*P. vivax*) vertical dotted lines represent the times when the treatment was administered. Figure shows mean(sem).

Among the eight volunteers studied in the *P. vivax* IBSM study, parasitaemia was first detected by PCR on Day 4 (95.4 h after innoculation), and peaked on the day of treatment (Day 10 in the morning = 244 h) at a mean of 232 (±49) parasites μl^−1^ (figure 3(A)). The course of parasitaemia observed is consistent with previous reports [15]. Seven of the 144 scheduled breath samples (18 time point collections × 8 volunteers) were not collected due to exacerbation of volunteers’ symptoms upon antimalarial treatment. The timing of missed samples is detailed in supplementary table 1.

Before treatment, thioethers were detected in the breath samples of subjects with experimental *P. vivax* infection (figure 3(A), supplementary figure 5),but the patterns and levels were different from those observed in *P. falciparum* IBSM. (i) As the *P. vivax* parasitaemia increased, the mean levels of MPTNZ, MPTNE and MTP in EBV remained low and did not approach those observed at comparable stages in the *P. falciparum* experiment (figure 3(B) and supplementary figure 6). For example, for MPTNZ, there was a 4.6 fold difference between the highest level in *P. falciparum* and the highest level in *P. vivax* infection. (ii) Systematic or consistent changes in the levels of thioethers were not observed in *P. vivax* volunteers (figure 3(B) and supplementary figure 6). (iii) Nor did the changes in levels of thioethers appear to be synchronized among individuals with *P. vivax* infection (supplementary figure 7). (iv) The thioethers MPTNE, AMS and MTP in the *P. vivax* volunteers were, on average, slightly above the levels of those of the controls before treatment (supplementary figure 5). However, the elevation was not statistically significant and, therefore, the thioethers could not be used to predict disease *P. vivax* infection.

### Several terpenes were identified in the breath of P. vivax and P. falciparum infection

In the absence of a notable thioether signal in *P. vivax* infection, we initiated an untargeted search for other volatiles associated with the course of *P. vivax* infection. A number of EBV features, i.e. unique combination of specific values of mass to charge ratio (*m/z*) and GC RT, were found to be associated with disease progression. Some features identified as informative shared similar retention times and were identified as fragments from the same compound. In total, seven potential volatile biomarkers for *P. vivax* infection (table 1) were identified. Four of these volatiles were fully identified using standards and shown to be terpenes, namely: α-terpinene, m-cymene, limonene and terpinolene. Three other volatiles could not be identified.

**Figure 4:**
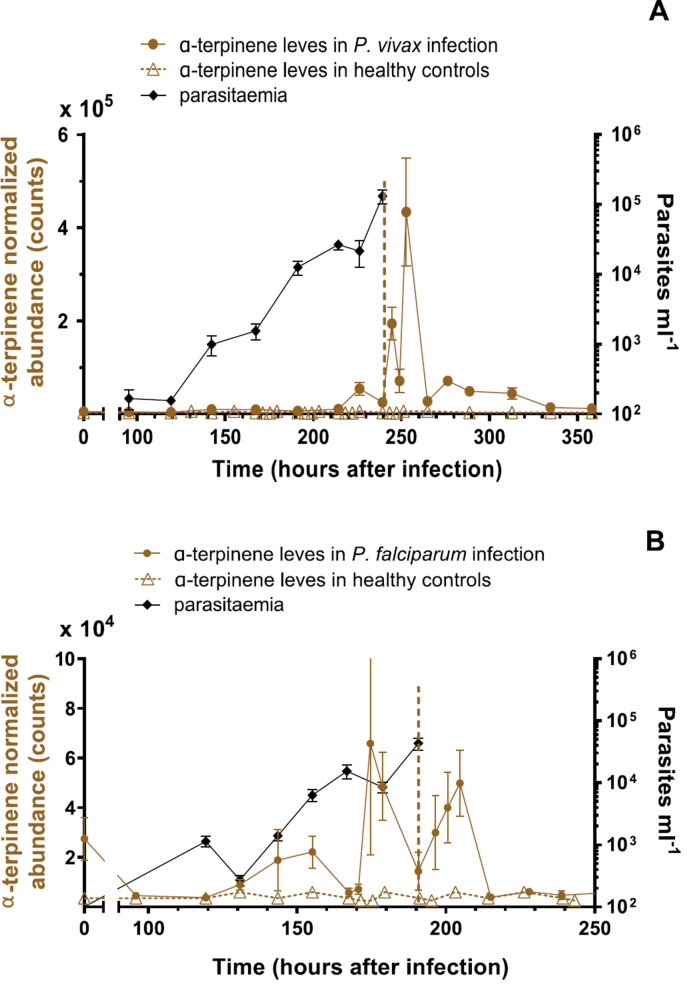
(A) Time course of α-terpinene abundance in the breath of CHMI *P. vivax* volunteers (*N* = 8), healthy controls (*N* = 8) and parasitaemia. (B) Time course of α-terpinene levels for *P. falciparum* CHMI trials (*N* = 7), healthy controls (*N* = 8) and parasitaemia. *P. vivax* and *P. falciparum* infections were started immediately after the breath sample at time zero. The vertical dotted lines represent the time when treatments were administered. The healthy control data shows the mean levels of the compound at that the nominated time of day, derived from three consecutive days of measurement. The diurnal cycle is repeated over the duration of the IBMS trial. The collection time matched to CHMI breath collections. Figure shows mean(sem).

Alpha-terpinene, m-cymene, limonene, terpinolene, ‘unknown 1’ and ‘unknown 2’ shared similar patterns of variation over the course of *P. vivax* infection and their levels rose as the infection progressed. Three noticeable increases and decreases wereobserved, with the largest peak 12 h after treatment, but we did not observe a regular periodicity (figure 4(A) and supplementary figure 8). It is important to note that we observed neither elevation nor cycling of well-known breath volatiles including isoprene and hexanal (supplementary figure 9 and supplementary table 5).

We are unaware of previous reports of an association between breath terpenes and *P. vivax* infection. Although it is not possible to perform *in vitro* culture of *P. vivax*, the terpenes identified in this study are similar to those previously reported in *P. falciparum* *in vitro* cultures [24] and *in vivo* [25]. We therefore investigated if the same terpenes present in the breath of volunteers infected with *P. vivax* are also present in breath samples of *P. falciparum* IBSM volunteers. We specifically searched for the features listed in table 1 and found that alpha-terpinene, m-cymene, limonene, terpinolene and ‘unknown 1’ and ‘unknown 3’ compounds were present in the breath samples. As in *P. vivax*, levels of α-terpinene in *P. falciparum* IBSM increased and decreased across the duration of malaria infection but with no regular periodicity (figure 4(B)).

Terpinolene, ‘unknown 1’, and m-cymene reached similar levels in breath of participants with *P. falciparum* to those observed in *P. vivax*. Limonene was also present in the breath of the *P. falciparum* studies, albeit at three-fold lower levels (95% CI: 2.3–3.58) than those found in *P. vivax*. The levels of all terpenes in the *P. falciparum* studies rose and fell in a similar fashion to that observed in *P. vivax* (figure 4(B), supplementary figure 10).

Other common breath volatiles such as isoprene neither cycled nor increased over the course of *P. falciparum*-infection (supplementary figure 11). The less abundant reference compound, hexanal, did not increase significantly over the course of malaria infection but did show circadian cycle before drug administration.

Machine learning methods were used to test our hypothesis that the terpene levels are significantly different between the breath of malaria-infected volunteers and healthy volunteers. As previously described in the methods section, SVM and k-NN classifiers were applied to the terpene levels. For *P. vivax* and *P. falciparum*-infected volunteers, we used all data (since there was no obvious cycling in these biomarkers) from Day 6 (155 h after collection) to just the time before treatment. The parasitaemia at the peak of infection, just before treatment, was 131 parasites μl^−1^ for *P. vivax* and 43 parasites μl^−1^ for *P. falciparum*. For healthy controls, we used data containing 48 random samples(see methods section for details).

For *P. vivax* and healthy controls, using leave-oneout cross-validation, all classification accuracy rates were above the statistically significance level (i.e. 58%, 56/96 samples). The best classification was achieved with terpinolene (91%), followed by m-cymene (75.8%) using SVM_L_. Both compounds showed 97.1% specificity and 54.6% and 85% sensitivities for terpinolene and m-cymene respectively. Similar results were found with the *P. falciparum*-infected samples and healthy control data. The top two compounds with the highest classification accuracies were terpinolene (87.7%), followedby m-cymene (92.7%). Average classification results for other compounds and classifiers can be found in supplementary table 6 with full results in supplementary data.

We trained the classifiers using *P. vivax* data and healthy data and they were able to classify *P. falciparum*-infected versus healthy control data for m-cymene and terpinolene, with 94% and 93% accuracies respectively (supplementary table 6). Although less accurate, when the classifier was trained with *P. falciparum* and the control data and applied to *P. vivax* data, terpinolene (79%) and m-cymene (75%) gave statistically significant levels of correct classification.

Untargeted search of biomarkers using *P. falciparum* data was also carried out but showed nothing of significance that warranted investigation.

## Discussions

In this study, we found that the previously reported thioether signal is characteristic of *P. falciparum*. The levels of thioethers show a pattern of cyclical variation. Their mean levels were significantly higher in the breath of *P. falciparum* IBSM volunteers compared with breath of healthy and P. vivax IBSM volunteers.

Our findings show that, when time of collecting breath samples is considered, accurate prediction of *P. falciparum*-infection can be achieved (98%) using the thioethers. Although validation in field samples and in a larger number of subjects with higher levels of parasitaemia is required, these results suggest that breath thioethers show potential as a diagnostic tool to screen individuals anddetect asymptomaticcases (>8.4 parasites μl^−1^). The detection limit for infection using these breath markers is lower than blood smear and RDT. Although an expert microscopist can detect as few as 5 parasites μl^−1^, an average microscopist detects only 50–100 parasites μl^−1^, similar to RDT which can detect between 50–100 parasites μl^−1^[26].

The diurnal cycling of breath volatiles has implications for the use of this approach to diagnose malaria, and suggests that the sensitivity of diagnostic breath testing may vary according to the time of day that the sampling is undertaken. Nocturnal or early evening breath sampling may be required to successfully detect falciparum malaria infection using the thioethers.

Thioethers have previously been found in the breath and blood of healthy individuals [27, 28] and we have now shown that their levels display a circadian rhythm in the breath of healthy volunteers suggesting that the compounds are of endogenous origin. Moreover, we previously established that thioethers were undetectable in the headspace above in vitro cultures of *P. falciparum*, even at high parasitaemia levels [8]. Considering these facts we hypothesize that the elevated levels of thioethers in breath of volunteers with *P. falciparum* are caused by the stimulation or enhancement of endogenous human biochemical pathways by the parasite. Cyclic elevation of thioethers was not observed in *P. vivax* infection, therefore it is likely that the thioethers are associated with unique factors in the pathology of *P. falciparum*.

In an untargeted search for biomarkers, we found that a rise in the level of terpenes in EBV occurs in both *P. vivax* and *P. falciparum* infections. Terpenes in exhaled breath: (i) increase in concentration and cycled over the course of malaria infection, (ii) peaked 12 h after drug treatment (figure 4(A) and supplementary figure 8), (iii) showed synchrony between individuals with respect to the variations in their levels (supplementary figure 12), (iv) included a terpene, limonene, that has previously been reported to be associated with *P. falciparum* in cell cultures [24] and to the isoprenoid precursor (E)-4-hydroxy-3-methylbut-2-enyl pyrophosphate (HMBPP) produced by *P. falciparum*, which indirectly triggers human red blood cells to increase the release of monoterpenes [29]. Recently, Schaber *et al* [25] found that two mosquito attracting terpenes, α-pinene and 3-carene, were present at significantly higher levels in the breath of children with malaria compared with uninfected children. (v) The four terpenes we identified in EBV of infected volunteers were either found at very low levels or were absent (e.g. ‘unknown 1’ and ‘unknown 2’ compounds) in the breath of healthy volunteers (figure 4(A) and supplementary figure 8). When present in breath of healthy individuals, terpenes showed no significant diurnal changes (supplementary figure 13). (vi) The infection status could be predicted, using a single terpene (SVM_L_ classifier), with up to 92% accuracy for *P. falciparum* and 91% for *P. vivax*.

Terpenes like α-pinene and limonene, were found specifically and consistently in the headspace of parasite-infected red blood cells [24]. The significance of this finding was that apicomplexan parasites, including Plasmodium species, contain an apicoplast (an organelle with a similar endosymbiotic evolutionary origin to plant chloroplasts) that synthesizes isoprenoids via the methylerythritol phosphate (MEP) pathway. This pathway is not present in animals. Isoprenoids serve multiple biochemical functions, including growth and development [30], asmembrane components, and as hormones [31]. Terpenes belong to the isoprenoid class. As postulated by Kelly *et al* [24], *Plasmodium falciparum* parasites might utilize the MEP pathway to produce terpenes. It has been recently been shown that two specific terpenes (αpinene and 3-carene) are elevated in the breath of children presenting with *P. falciparum* malaria to a clinic in Malawi[25].

There have beena number ofstudies onchanges in human odor due to malaria infection and how infection can affect mosquito behavior [32, 33]. One study examined the foot odor of CHMI volunteers (with low densities of a sexual parasites and no gametocytes), the carbonyls 2- and 3-methylbutanal, 3-hydroxy-2-butanone and 2-ethyl hexanoic acid were altered during and after CHMI [32]. Recently, Robinson *et al* [33] found that higher levels of six compounds, most of them aldehydes, were exhaled from children’ socks during natural malaria infection compared to the socks of parasite-free individuals. These aldehydes were positively correlated with overall parasite density and the blend of aldehydes increased the attractiveness to the malaria vector Anopheles coluzzii when tested with a synthetic mosquito lure. Moreover, another recent study also documented volatile changes in the skin (foot and arm) from children with asymptomatic and symptomatic malaria infections [34]. They found a set of malaria diagnostic volatiles coming from diverse chemical families, including aldehydes. None of these studies found the thioethers nor the terpenes, suggesting that parasite densities and developmental stage might induce a range of physiological changes in the human body that affect breath and body odor in variedways.

The data presented in this paper show diurnal cycling of thioethers in *P. falciparum* infection and represent the first evidence that EBV terpene levels increase in human *P. vivax* infection. These findings need to be tested in the field. If confirmed, terpenesmay be more convenient as a malaria biomarker because of their lack of diurnal variation. However, the prediction power of using terpenes and thioethers should also be considered. It would bedesirable also to understand the impact of mixed infections on the levels of both thioethers and terpenes in human breath.

## Research data

The raw chromatographic data used in this paper is available online[35–37].
