# Supplementary material for: Diurnal variation in expired breath volatiles in malaria-infected and healthy volunteers
Source: J Breath Res. 2018 Sep 19;12(4):046014. doi: 10.1088/1752-7163/aadbbb (PMC7753889; doi:10.1088/1752-7163/aadbbb)
Supplement: Supplementary file 1 [file JBR-12-04-046014-s001.pdf]

SUPPLEMENTARY MATERIAL

Section S1. Additional Figures

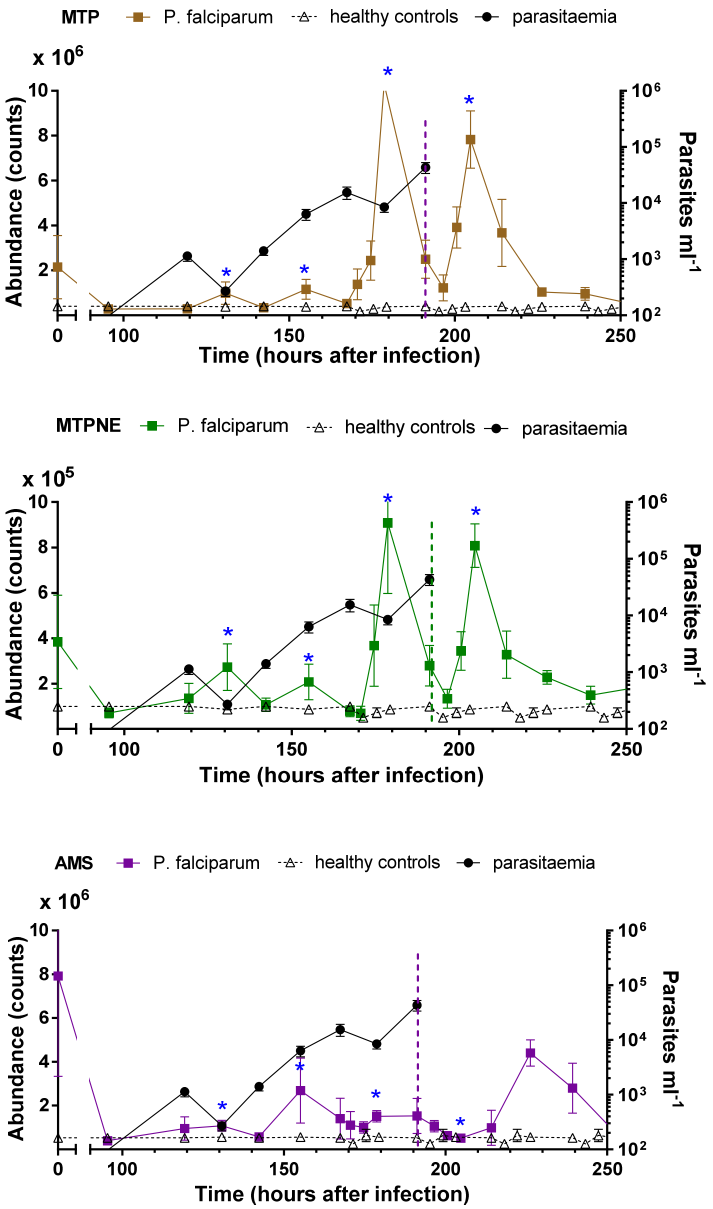

Supplementary Figure 1: Time course of mean levels of thioethers for *P. falciparum* CHMI trials (N=7), healthy controls (N=8) and parasitaemia. *P. falciparum* infection started immediately after the breath sample at time zero. The vertical dotted line represents the time when treatment was administered in *the P. falciparum* trial. The healthy control data shows the means of three consecutive days of measurements. The diurnal cycle is repeated over the duration of the CHMI trial and the collection time matched as closely as possible to CHMI collections. Figure shows mean(sem). Sem is used as a measure of precision for the estimated mean. Samples with asterisks in blue denote sampling time between 19:00 and 21:00. MTP= 1-methylthio-propane, MTPNE = (E)-1-methylthio-1-propene and AMS = allyl methyl sulphide.

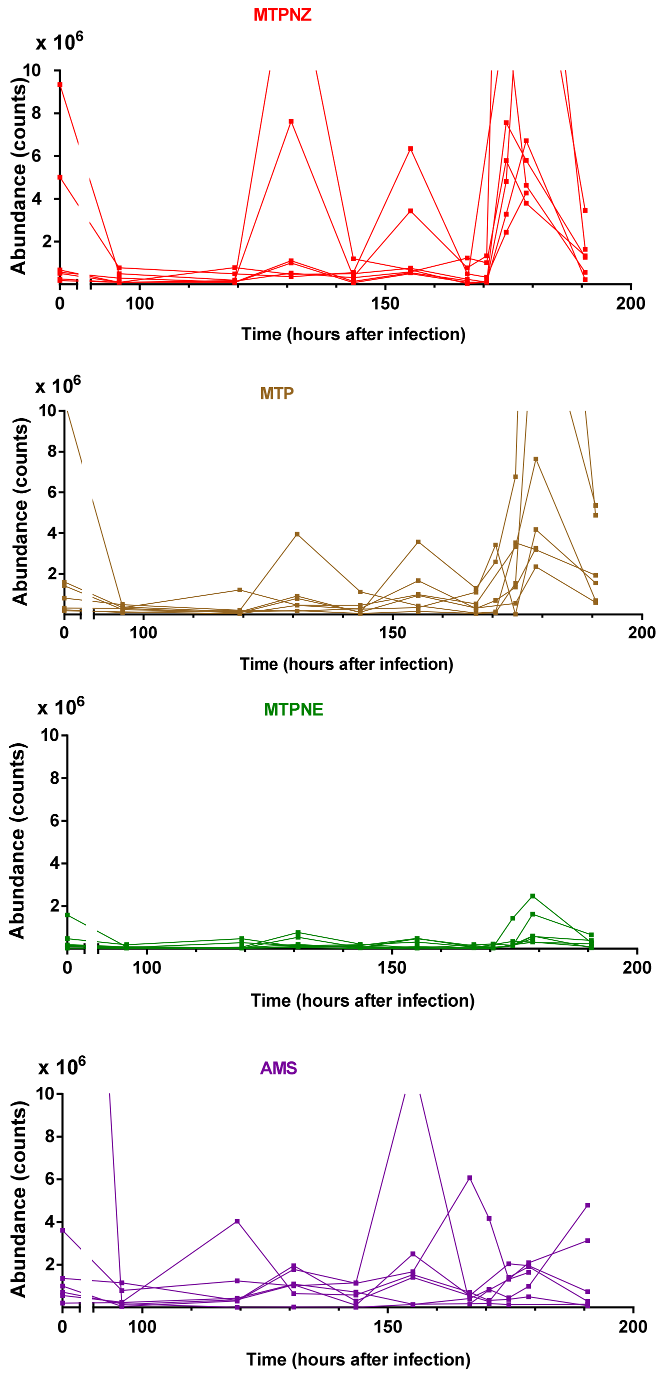

Supplementary Figure 2: Thioether levels for individual volunteers in the *P. falciparum* CHMI trial (N=7)**.** MTPNZ=(Z)-1-methylthio-1-propene, MTP= 1-methylthio-propane, MTPNE = (E)-1-methylthio-1-propene and AMS = allyl methyl sulphide.

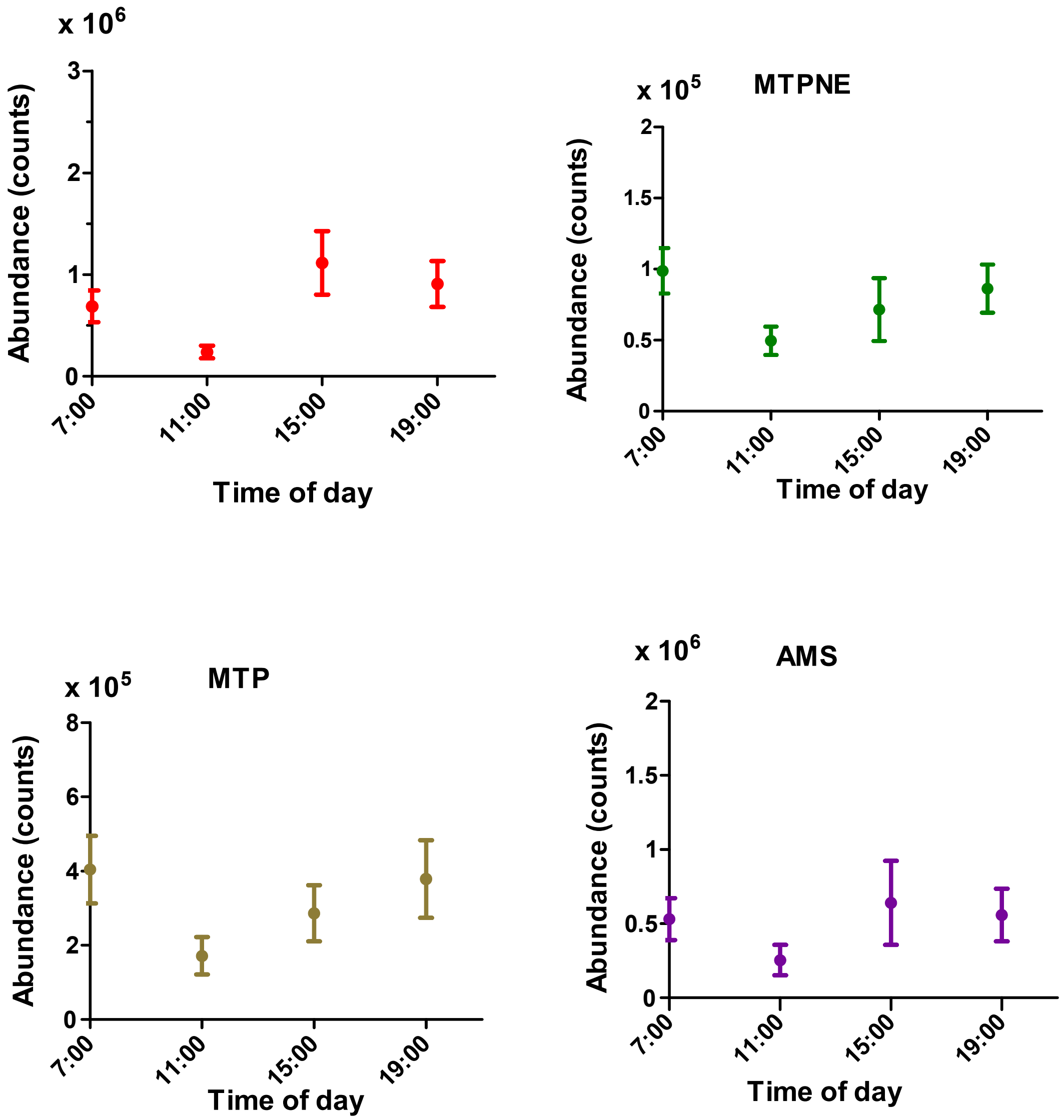

Supplementary Figure 3: Average concentrations of thioethers in the breath of healthy individuals. Data collected over three consecutive days and for N=8 individuals. Figure shows mean(sem). MTPNZ=(Z)-1-methylthio-1-propene, MTP= 1-methylthio-propane, MTPNE = (E)-1-methylthio-1-propene and AMS = allyl methyl sulphide

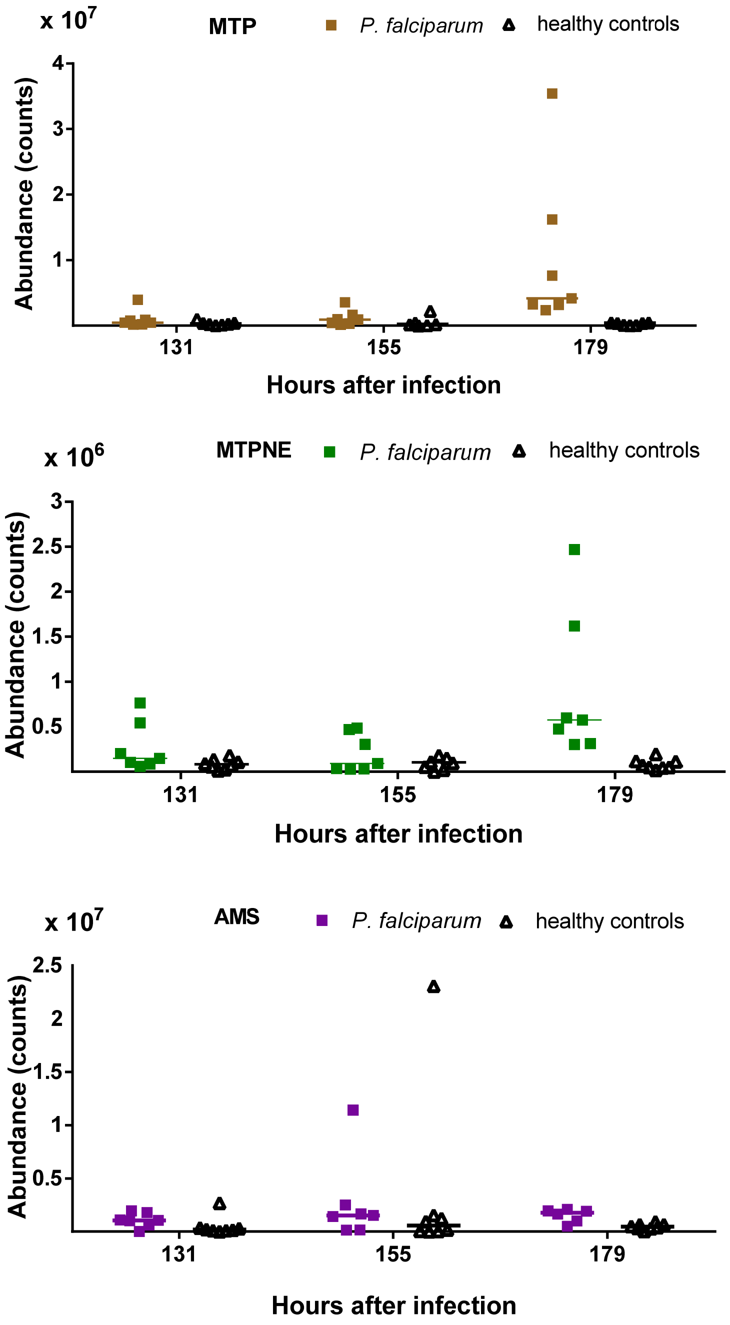

Supplementary Figure 4: Comparisons of mean thioethers levels for *P. falciparum* (N=7) CHMI trial and healthy controls (N=8) for samples collected between 19:00 and 21:00 hours. For the CHMI trial, the plot shows breath samples collected at 131 hours (Day 5), 155 hours (Day 6) and 179 hours (Day 7) after infection., MTP= 1-methylthio-propane, MTPNE = (E)-1-methylthio-1-propene and AMS = allyl methyl sulphide.

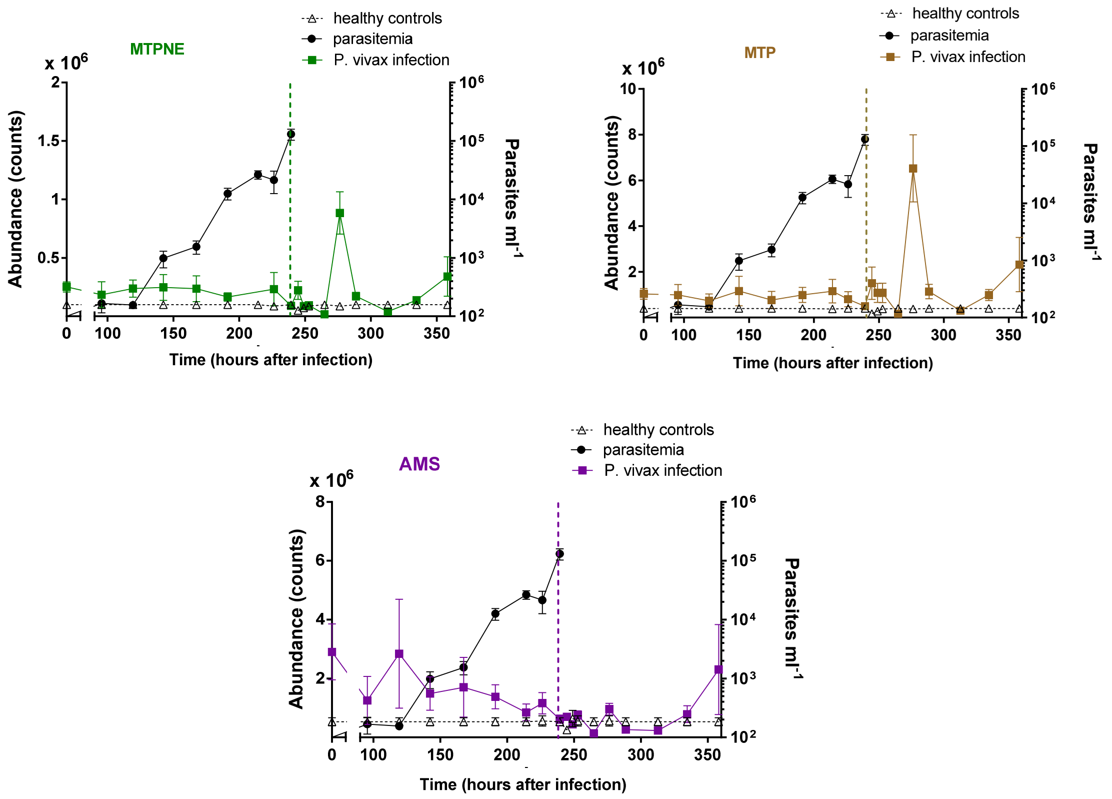

Supplementary Figure 5: Time course of thioethers in breath of participants experiencing CHMI with *P. vivax* (N= 8), healthy controls (N=8) and parasitaemia levels. Vertical dotted line represents the time when experimental antimalarial was administered. The healthy control data shows the mean levels of the compound found at that time of day, derived from three consecutive days of measurements. The diurnal cycle is repeated over the duration of the IBSM trial, and the collection time matched as closely as possible to collections from the P. falciparum-infected subjects. Figure shows mean(sem). SEM is used as a measure of precision for the estimated mean. MTP= 1-methylthio-propane and MTPNE = (*E*)-1-methylthio-1-propene and AMS = allyl methyl sulphide.

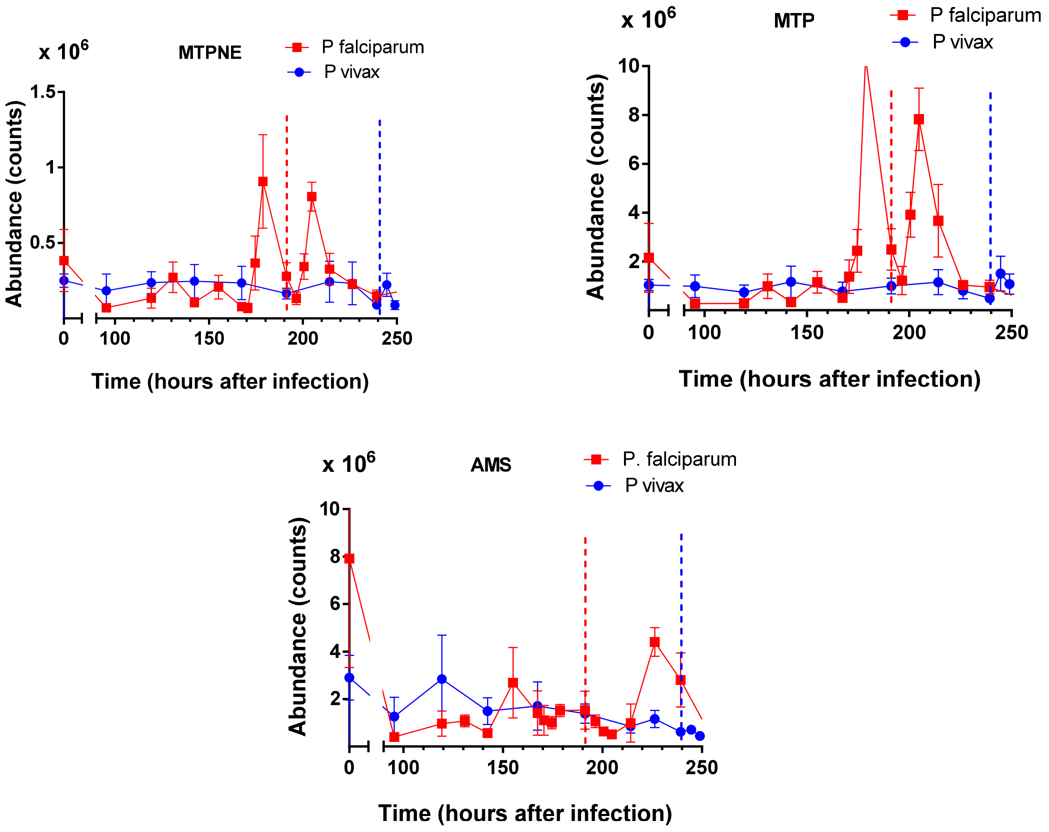

Supplementary Figure 6: Comparisons of mean of thioether levels for *P. falciparum* (N=7) and *P. vivax* (N=8) CHMI trials. Both infections were initiated immediately after the baseline breath sample, taken at time zero. Red (*P. falciparum*) and blue (*P. vivax*) vertical dotted lines represent the times when the treatment was administered. Figure shows mean(sem). MTP= 1-methylthio-propane and MTPNE = (*E*)-1-methylthio-1-propene and AMS = allyl methyl sulphide

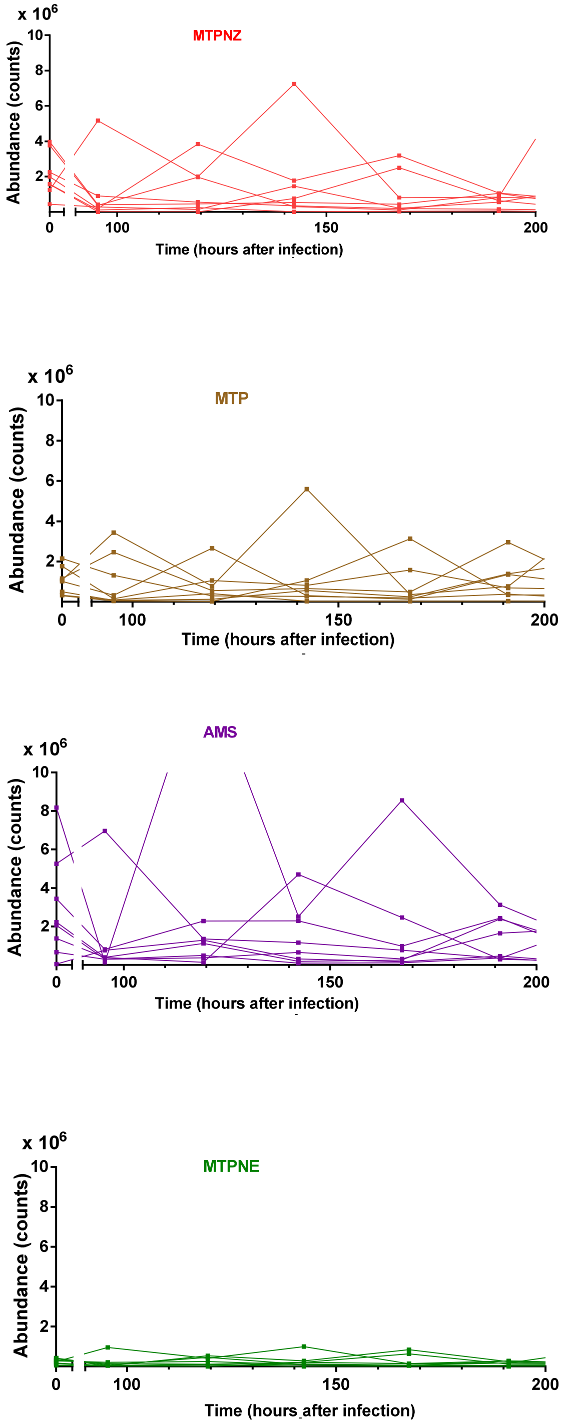

Supplementary Figure 7: Thioether levels for individual volunteers in the *P. vivax* CHMI trial (N=8). MTP= 1-methylthio-propane, AMS = allyl methyl sulphide, MTPNZ = (Z)-1-methylthio-1-propene and MTPNE = (E)-1-methylthio-1-propene.

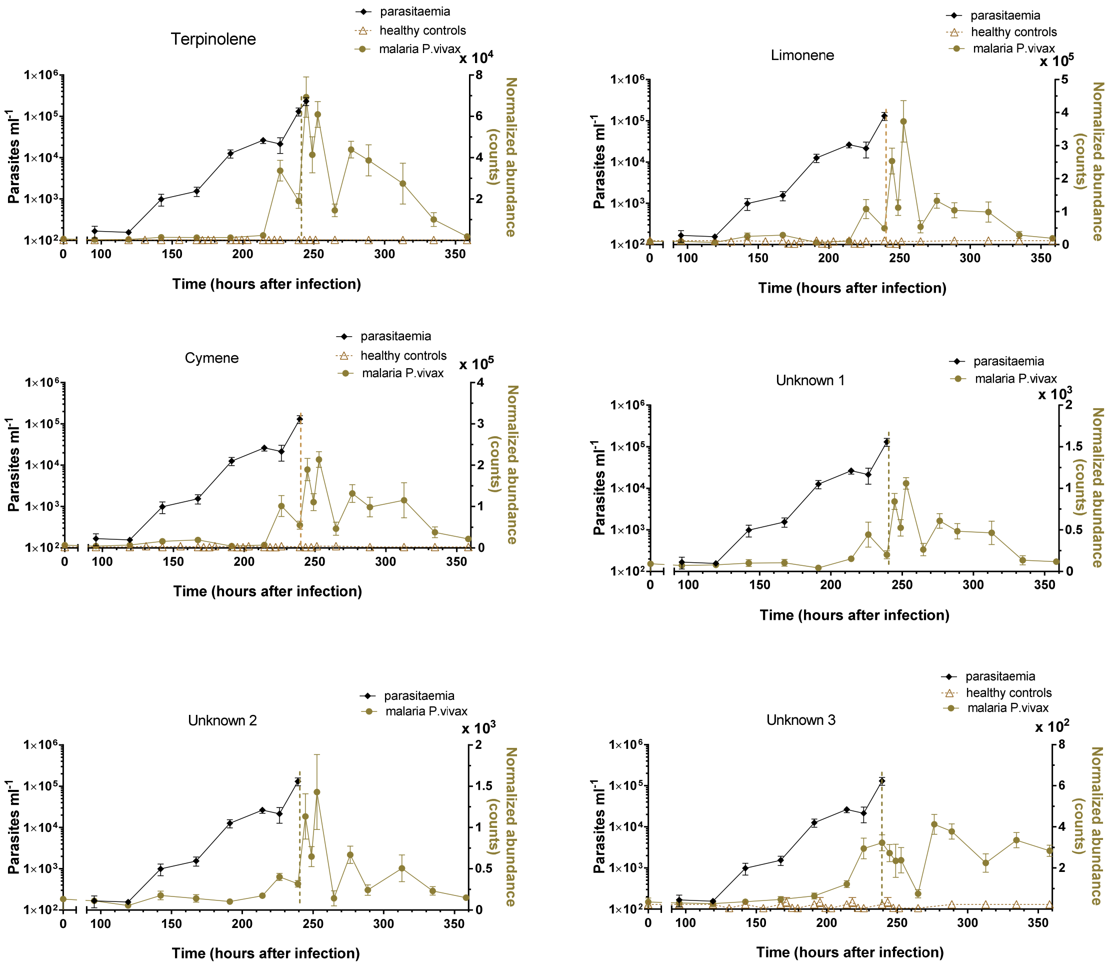

Supplementary Figure 8: Time course of terpenes and unknowns for *P. vivax* CHMI trials (N=8), healthy controls (N=8) and parasitaemia. *P. vivax* infection started immediately after the breath sample at time zero. The vertical dotted line represents the time when treatment was administered. The healthy control data shows the means of three consecutive days of measurements. The diurnal cycle is repeated over the duration of the CHMI trial and the collection time matched as closely as possible to CHMI breath collections. Figure shows mean(sem).

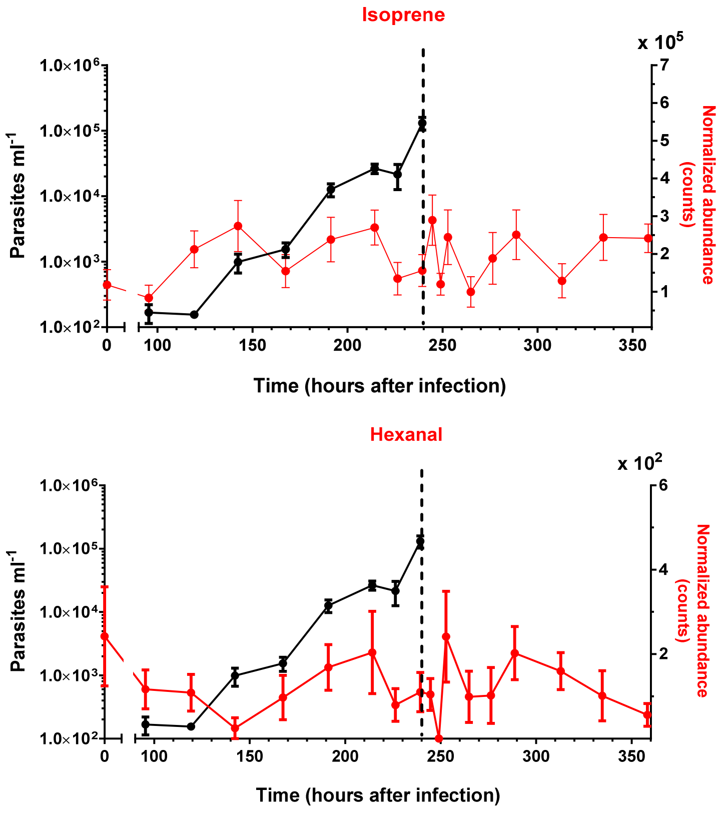

Supplementary Figure S9: Time course of isoprene and hexanal for *P. vivax* CHMI trials (N=8) and parasitaemia. *P. vivax* infection started immediately after the breath sample at time zero. Retention time and *m/z* used for these plots are shown in Table S5. The vertical dotted line represents the time when treatment was administered. Figure shows mean(sem).

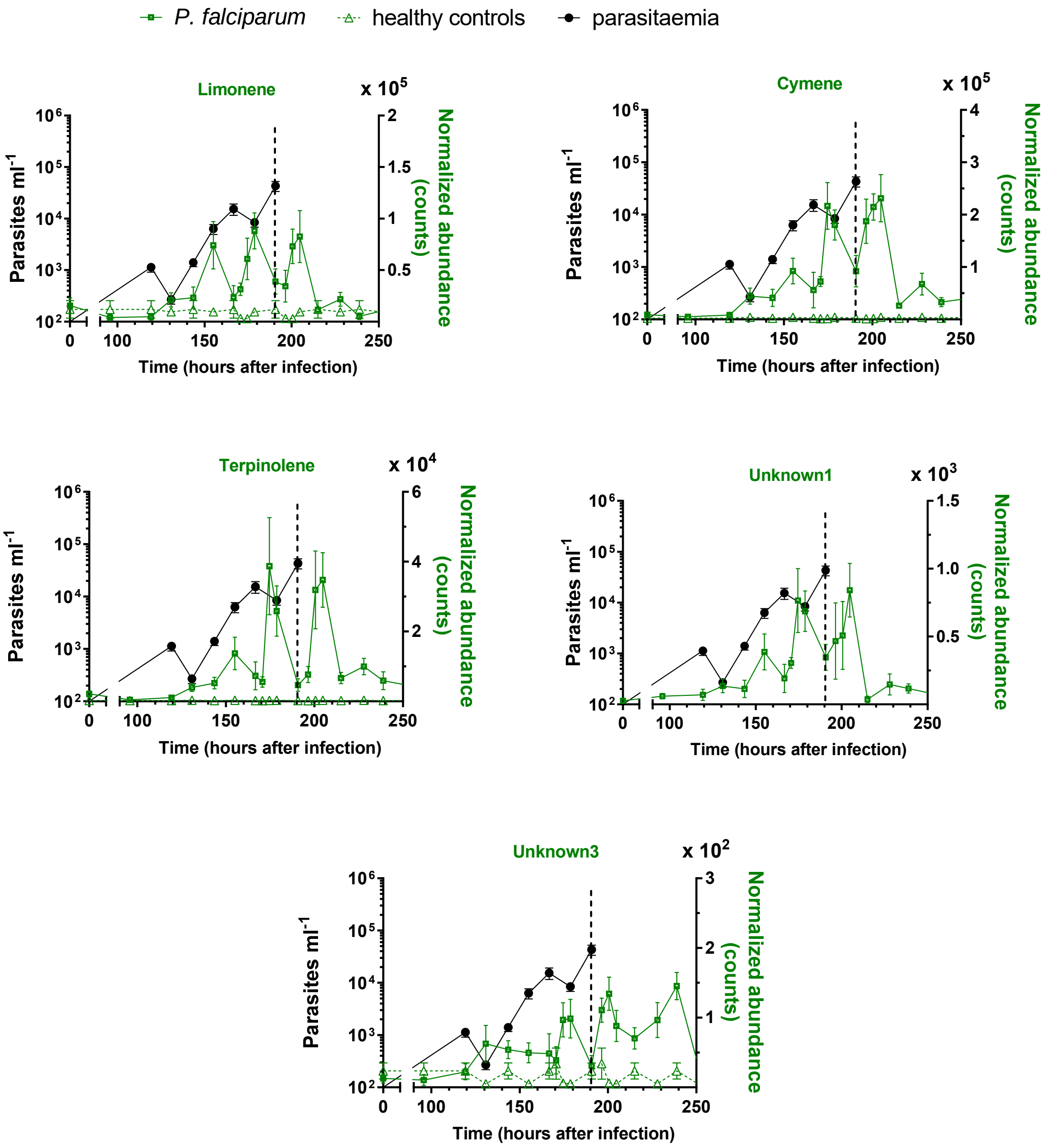

Supplementary Figure 10: Time course of terpenes and unknowns for *P. falciparum* CHMI trials (N=7), healthy controls (N=8) and parasitaemia. *P. falciparum* infection started immediately after the breath sample at time zero. The vertical dotted line represents the time when treatment was administered. The healthy control data shows the means of three consecutive days of measurements. The diurnal cycle is repeated over the duration of the CHMI trial and the collection time matched as closely as possible to CHMI breath collections. Figure shows mean(sem).

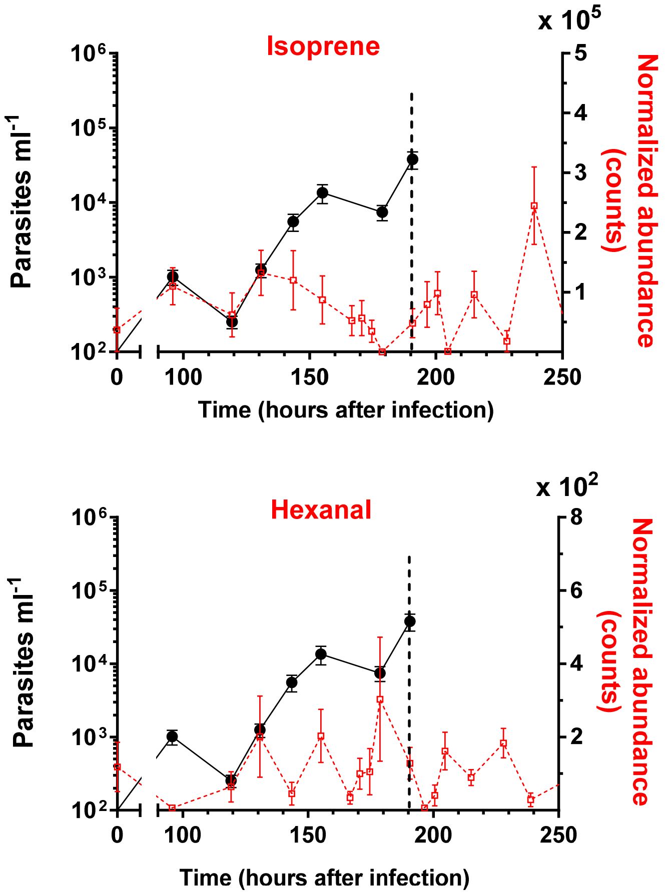

Supplementary Figure 11: Time course of isoprene and hexanal for *P. falciparum* CHMI trials (N=7) and parasitaemia. *P. falciparum* infection started immediately after the breath sample at time zero. Retention time and m/z used for these plots are shown in Table S5. The vertical dotted line represents the time when treatment was administered. Figure shows mean(sem).

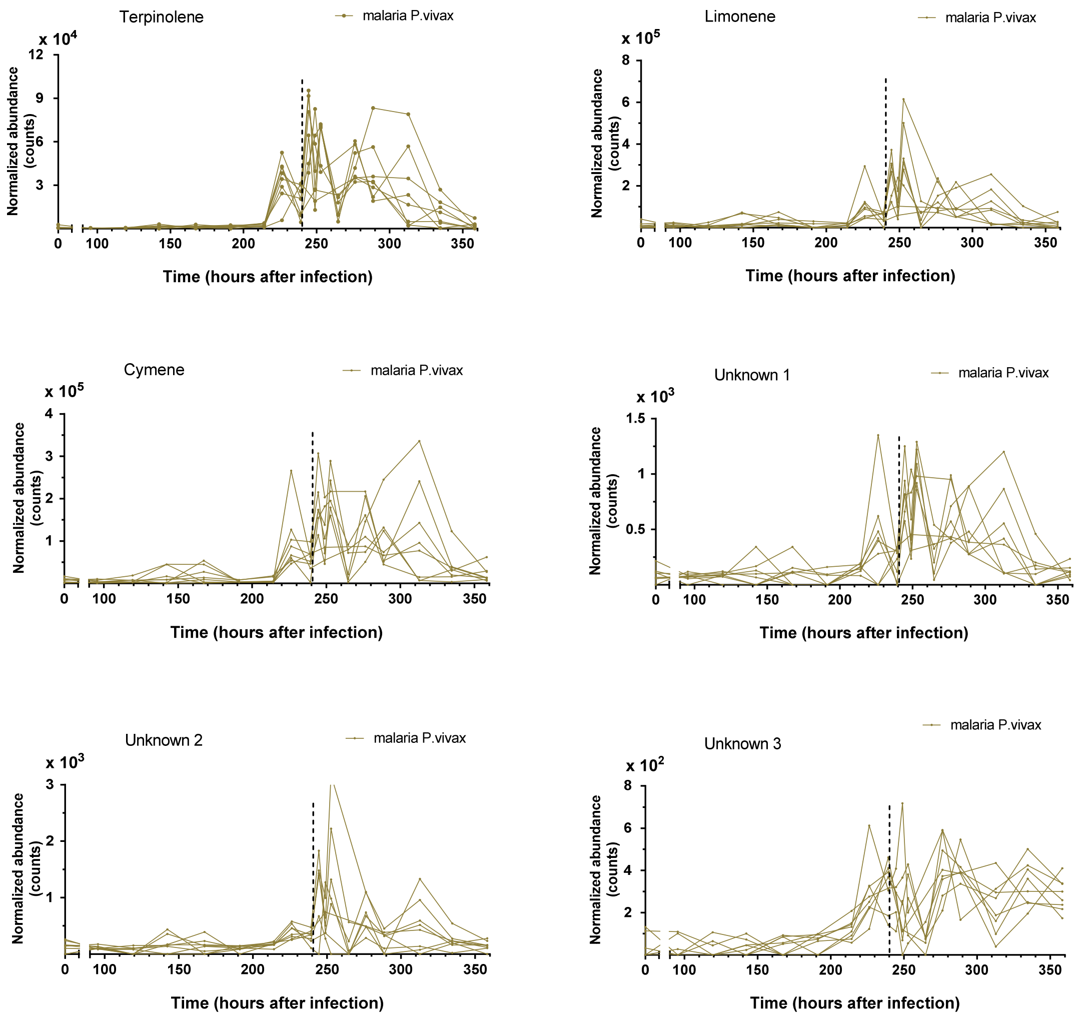

Supplementary Figure 12: Time course of terpene levels and unknowns for individual volunteers in the *P. vivax* trial (N=8)**.** Vertical dotted lines represent the time when the treatment was administered.

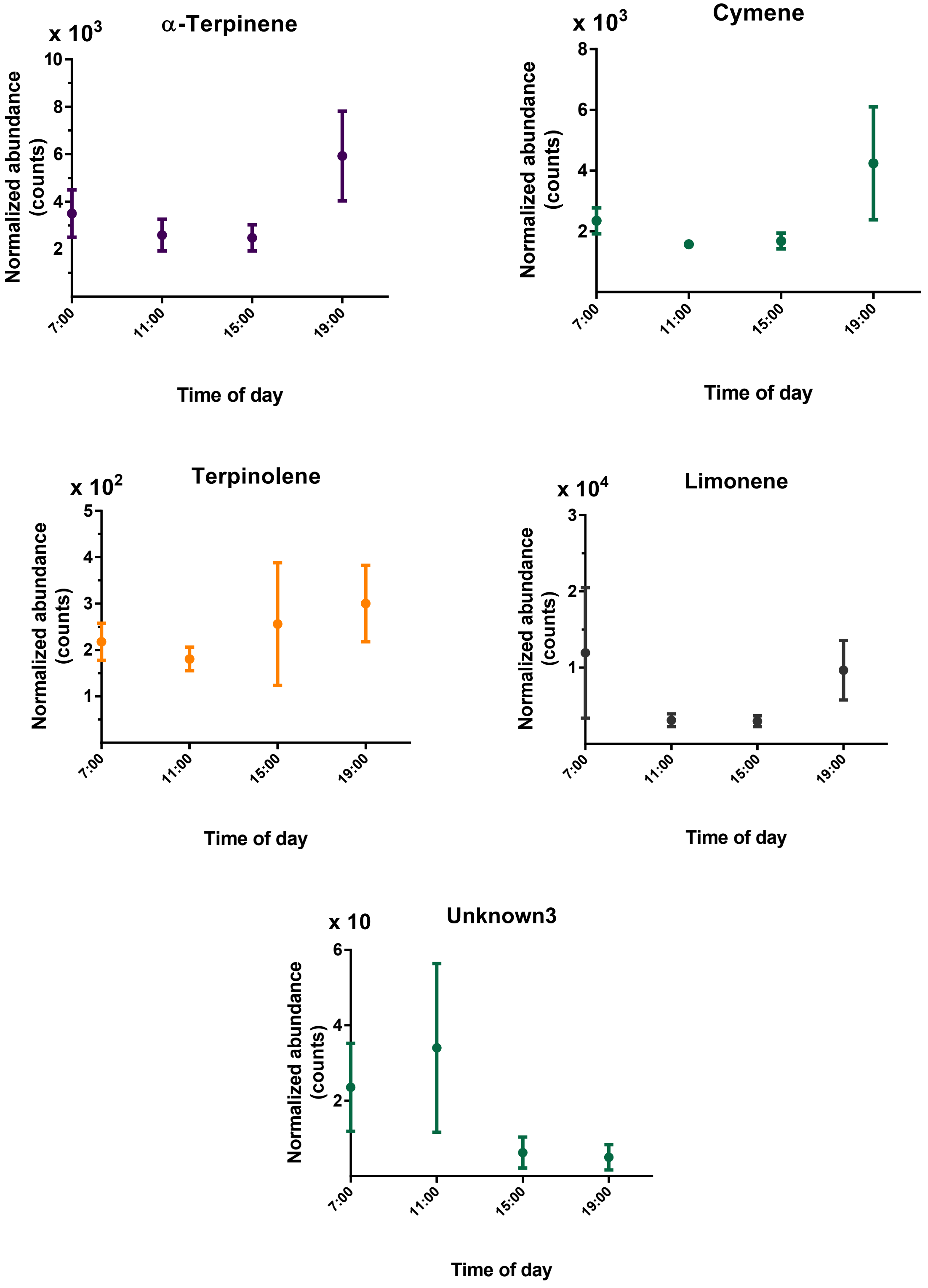

Supplementary Figure 13: Diurnal changes of terpenes and unknown3 concentrations in healthy individuals. Data collected over three consecutive days and for N=8 individuals. Figure shows mean(sem).

Supplementary Table 1A. Breath samples collected and analyzed over malaria course for the *P. falciparum* and *P. vivax*.

Cohort	Day 0	Day 4	Day 5	Day 6	Day 7	Day 8	Day 9	Day 10	Day 11	Day 12	Day 13	Day 14	Day 15	Total time points collected
P. falciparum	X	X	X[¥]	X[¥]	X[ε]	X[εß]	X[¥]	X[¥]						18
P. vivax	X	X	X	X	X	X	X[¥]	X[εß] (4)[§]	X[¥] (3)[§]	X	X	X	X	18

X=Days of breath sample collection
Day 0=Malaria parasite inoculum
¥ =indicates that two breath samples were collected per volunteer: one sample in the morning (around 7:00) and a second sample 12 hours later
ε = indicates that four breath samples were collected per volunteer: around 7:00, 11:00, 15:00 and 19:00.
ß = indicates day of admission
§=number of breath samples missed due to inability of volunteer to provide breath samples
Confinement phase=Day 8, 9 and 10 for *P. falciparum* and Day 10, 11 and 12 for *P. vivax*

Supplementary Table 1B. Days of breath samples collected in eight healthy volunteers

Participant No.	Week 1			Week 2			Week 3		
	Day 1	Day 2	Day 3	Day 1	Day 2	Day 3	Day 1	Day 2	Day 3
1	X	X	X						
2	X	X	X						
3	X	X	X						
4				X	X	X			
5				X	X	X			
6				X	X	X			
7							X	X	X
8							X	X	X

X=Days of breath sample collection. In each day 4 samples were collected, time points were 7:00, 11:00, 15:00 and 19:00

Supplementary Table 1C. Number of breath samples collected per healthy volunteer and per collection time point over three days of collection

Participant No.	7:00	11:00	15:00	19:00	Total
1	3	3	3	3	12
2	3	3	3	3	12
3	3	3	3	3	12
4	3	3	3	3	12
5	3	3	3	3	12
6	3	3	3	3	12
7	3	3	3	3	12
8	3	3	3	3	12
No. of samples per time point	24	24	24	24	96

Supplementary Table 2: Mean, standard error of the mean (SEM) and multicomparison test for the thioethers in healthy controls at different time of day

Thioethers	Time of day	Mean* (counts)	SEM	Multicomparison (Bonferroni correction)**
MTPNZ	7:00	6.88e+05	1.55e+05	11:00 vs 15:00 (p=0.0028)<br>11:00 vs 19:00 (p=0.028)
	11:00	2.39e+05	6.25e+04	
	15:00	1.11e+06	3.11e+05	
	19:00	9.08e+05	2.26e+05	
MTP	7:00	4.04e+05	9.05e+04	7:00 vs 11:00 (p=0.007)<br>11:00 vs 19:00 (p=0.019)
	11:00	1.71e+05	5.02e+04	
	15:00	2.86e+05	7.56e+04	
	19:00	3.78e+05	1.04e+05	
MTPE	7:00	9.88e+04	1.60e+04	7:00 vs 11:00 (p=0.0141)
	11:00	4.95e+04	9.94e+03	
	15:00	7.15e+04	2.21e+04	
	19:00	8.63e+04	1.69e+04	
AMS	7:00	5.31e+05	1.41e+05	n.s.
	11:00	2.54e+05	1.01e+05	
	15:00	6.40e+05	2.84e+05	
	19:00	5.58e+05	1.77e+05	

*Data represents the mean over the 3 days of experiment.
**Results shown only for pairs whose mean difference is significant at 0.05 level.
n.s.= no significant differences found between pairs

Supplementary Table 3: Mean, standard error of the mean (SEM), fold change (95% CI) and p-value results at the peak of thioether evolution for *P. falciparum* CHMI volunteers

Thioether	Hours after treatment	Mea n± SEM *P. falciparum*	Mean ± SEM* Healthy controls	Fold change (95% CI)	p-value
MTPNZ	131 (Day 5)	3.85E+06 ± 2.24E+06	1.02E+06 ± 3.65E+05	3.77 (-1.04 – 19.80)	0.20
	155 (Day 6)	1.84E+06 ± 8.52E+05	6.21E+06 ± 4.96E+06	0.30 (**)	0.43
	179 (Day 7)	1.10E+07 ± 4.11E+06	5.32E+05 ± 1.58E+05	20.68 (3.81 - 66.48)	**0.02**
MTP	131 (Day 5)	9.86E+05 ± 5.04E+05	3.59E+05 ± 1.15E+005	2.75 (-0.29 - 10.83)	0.22
	155 (Day 6)	1.15E+06 ± 4.46E+05	5.17E+05 ± 3.51E+05	2.22 (**)	0.28
	179 (Day 7)	1.03E+07 ± 4.55E+06	2.77E+05 ± 7.01E+04	37.18 (1.69 - 104.39)	**0.03**
MTPE	131 (Day 5)	2.72E+05 ± 1.02E+05	8.58e+04 ± 1.97E+04	3.17 (0.59 - 7.82)	0.08
	155 (Day 6)	2.07E+05 ± 7.88E+04	4.64E+05 ± 3.77E+05	0.45 (**)	0.54
	179 (Day 7)	9.07E+05 ± 3.10E+05	8.32E+04 ± 2.03E+04	10.90 (2.72 - 27.47)	**0.01**
AMS	131 (Day 5)	1.07E+06 ± 2.47E+05	4.93E+05 ± 3.14E+05	2.17 (**)	0.18
	155 (Day 6)	2.68E+06 ± 1.48E+06	3.38E+06 ± 2.82E+06	0.79 (**)	0.84
	179 (Day 7)	1.51E+06 ± 2.58E+05	4.735E+05± 9.94E+04	3.19 (1.77 - 6.26)	**0.00**

*For the healthy controls the three days of measurements shown are means of samples collected at around 19:00 hours on Day 1, Day 2 and Day 3 respectively.
(**) Since the confidence interval of the denominator includes zero, it is not possible to compute the CI of the quotient.
Number in in bold denote differences are statistically significant

Supplementary Table 4A: Average accuracy for malaria diagnosis using two classifiers (SVM and k-NN). Samples used for classification are those collected between 19:00 and 21:00 and before treatment for the *P. falciparum* CHMI trial.

Day 5 (131 hours after infection)

Compound	SVML	kNN3
AMS	64.29%	71.43%
MTP	52.86%	35.71%
MPTN (E)	51.43%	47.14%
MTPN (Z)	57.14%	57.14%

Day 6 (155 hours after infection)

Compound	SVML	kNN3
AMS	**75.71%**	62.86%
MTP	45.71%	42.86%
MPTN (E)	68.57%	**80.00%**
MTPN (Z)	17.14%	60.00%

Day 7 (179 hours after infection)

Compound	SVML	kNN3
AMS	68.57%	68.57%
MTP	**98.57%**	**98.57%**
MPTN (E)	**91.43%**	**91.43%**
MTPN (Z)	**94.29%**	**94.29%**

Two classes: *P. falciparum* malaria samples (N=7) and healthy controls (N=7)
For healthy controls, the results presented are the average of five randomly selected data sets containing N=7 random breath samples.
Key: AMS= allyl methyl sulphide, MTP= 1-methylthio-propane, MTPNE= (*E*)-1-methylthio-1-propene and MTPNZ= (*Z*)-1-methylthio-1-propene.

The accuracy of the classification to predict the sample group was considered significant when the accuracy result was above the significance level at 95% confidence interval, i.e. 71.43% (10/14). Classification accuracy rates below the significance level value were considered non-significant. Numbers in bold means classification was above statistical significance levels (>71.43%).

Supplementary Table 4B: Average total number of correctly classified samples, sensitivity and specificity for those whose classifications were above the statistical level (p=0.05, 10/14) in Table 4A.

Day after infection	Compound	Total number of samples correctly classified/ total number of samples	Sensitivity	Specificity
Day 6 (155 hours after infection)	AMS	10.6/14	71.4%	80.0%
	MTPN (E)	11.2/14	71.4%	88.6%
Day 7 (179 hours after infection)	MTP	13.8/14	100%	97.1%
	MPTN (E)	12.8/14	82.9%	100%
	MTPN (Z)	13.2/14	100%	88.6%

Supplementary Table 5: Features (retention time (Rt) and mass to charge ratio (*m/z*)) used for semi quantification of isoprene and hexanal in healthy, *P. vivax* and *P. falciparum* samples (1).

Rt (min)	Mass to charge ratio (*m/z*)	Compound name
2.50	67	Isoprene
16.67	82	Hexanal

Supplementary Table 6: Average classification results for the terpenes using two classifiers in *P. falciparum* and *P. vivax* vs healthy controls data.

P. falciparum vs. healthy

	SVM_L			KNN 3		
Compound	accuracy	sensitivity	specificity	accuracy	sensitivity	specificity
alpha terpinene	69.58	49.58	89.58	73.13	71.67	74.58
Limonene	81.46	70.83	92.08	74.38	86.25	62.50
cymene	92.71	87.50	97.92	96.67	99.17	94.17
terpinolene	87.71	76.67	98.75	93.54	89.58	97.50

P. vivax vs. healthy

	SVM_L			KNN-3		
Compound	accuracy	sensitivity	specificity	accuracy	sensitivity	specificity
alpha terpinene	75.63	62.08	89.17	77.71	80.00	75.42
Limonene	72.71	53.75	91.67	73.96	87.92	60.00
cymene	75.83	54.58	97.08	74.58	69.58	79.58
terpinolene	91.04	85.00	97.08	73.54	85.83	61.25

P. falciparum as training

	SVM_L			KNN-3		
Compound	accuracy	sensitivity	specificity	accuracy	sensitivity	specificity
alpha terpinene	74.11	59.58	88.94	75.58	85.42	65.53
Limonene	74.53	59.17	90.21	72.00	83.33	60.43
cymene	74.74	50.00	100	77.26	60.42	94.47
terpinolene	78.95	59.17	99.15	90.32	82.92	97.87

P. vivax as training

	SVM_L			KNN-3		
Compound	accuracy	sensitivity	specificity	accuracy	sensitivity	specificity
alpha terpinene	72.00	57.50	86.81	71.37	73.33	69.36
Limonene	80.84	72.92	88.94	73.05	89.17	56.60
cymene	94.32	93.75	94.84	83.58	93.75	73.19
terpinolene	92.84	89.58	96.17	74.53	93.75	54.89

Section S2. Detailed methods

S2.1 *P. falciparum* and *P. vivax* trials

Ethics. This study was carried out as an adjunct to ongoing studies investigating novel or improved treatment regimes for malaria. The results from the antimalarial studies will be published elsewhere. Breath studies were conceived as an "add-on" to those pre-existing treatment studies. The entire study was approved by the Queensland Institute of Medical Research Human Research Ethics Committee (QIMR-HREC) (P1479) and endorsed by the CSIRO Health and Medical Research, Human Research Ethics Committee (proposal numbers: 11/2016 and 19/2016). The study was conducted in accordance with the Declaration of Helsinki principles for the conduct of clinical trials and the International Committee of Harmonization Good Clinical Practice Guidelines as recognized by the Australian Therapeutic Goods Administration (TGA). Written informed consent was received from participants prior to inclusion in the study. In *P. falciparum* trial, seven volunteers were recruited for the drug study all of whom agreed to provide breath. In the *P. vivax* trial, eight volunteers were recruited for breath collection.

Preparation of inocula. The production of the parasite inoculum has been described previously (2). In brief, laboratory-reared *Anopheles stephensi* mosquitoes were infected by membrane feeding on a blood meal containing gametocytes with the chloroquine-sensitive *P. falciparum* clone 3D7, derived from an isolate originally collected from an airport worker in Amsterdam. Ten days after their last blood meal, the mosquitoes were allowed to feed on a healthy male who had no evidence of any blood borne virus infection. A 500 mL unit of blood was taken from the volunteer six hours after he became ill with the development of high fever, 13 days after the mosquito bites. The blood was leucocyte depleted, mixed with the cryopreservation agent Glycerolyte 57, aliquoted into 1-mL cryovials and stored in liquid nitrogen at QIMR.

To prepare inocula for experimental infection studies, aliquots of the *P. falciparum* cell bank were thawed, washed, and resuspended in injectable saline solution according to a method described elsewhere (3). Parasites were synchronous. It was planned that each injected inoculum would contain ~120,000 erythrocytes, of which ~5,400 were parasite-infected. Each challenge inoculum was dispensed into syringes and stored in sealed plastic bags on ice until administered. The time between thawing and injection was no more than 60 minutes.

For *P. vivax*, aliquots of the *P. vivax* cell bank were thawed, washed, and resuspended in injectable saline solution according to a method described elsewhere (4), with the modification that the final red cell pellet was resuspended in normal saline solution rather than Roswell Park Memorial Institute medium. One mL of the resuspended cells was set aside for quantification of parasitaemia by polymerase chain reaction (PCR), and the remainder of the dose dispensed aseptically into a 2-mL syringe and stored on ice until administration. The number of parasite genome equivalents injected into subjects was determined using the quantitative PCR method described below.

Cohorts. Each participant in the *P. falciparum* and *P. vivax* was inoculated intravenously on Day 0 with ~1,800 viable *Plasmodium falciparum*-infected human erythrocytes and ~1,800 viable *Plasmodium vivax*-infected human erythrocytes. From Day 4 onwards, participants

were monitored daily for adverse events and for the unexpected early onset of symptoms, signs or parasitological evidence of malaria.

On the day designated for commencement of treatment, as determined by qPCR results, participants were admitted to the study unit and confined for safety monitoring and antimalarial drug administration, with parasite load and drug levels being monitored. The threshold for commencement of treatment was when PCR quantification was confirmed to be ≥ 1,000 parasites mL^{-1}. If clinical or parasitological evidence of malaria (the onset of clinical features of malaria) occurred, or if ≥1,000 parasites mL^{-1} was detected by PCR before the morning of Day 7, allocated treatment was started immediately.

Following treatment with an antimalarial, participants were followed up as inpatients for at least 48 hours, to ensure they tolerated the therapy and to confirm a clinical response then, if clinically well, on an outpatient basis for safety and to monitor for persistence of malaria parasites using PCR. Early intervention with Riamet® was planned if either poor responses or fast responses were seen following initial treatment with experimental drugs, to ensure patient safety.

PCR quantification of *Plasmodium* parasitaemia. A consensus Plasmodium species RT-PCR method described elsewhere (5) was modified to make use of TaqMan hydrolysis probe chemistry. The assay amplifies a conserved 199–base pair (bp) target in the multicopy, highly conserved, 18S ribosomal RNA gene. Parasites were quantified from 500 µL of packed red cells. Each sample was tested in duplicate during the study. After completion of the study, all samples were retested in triplicate. When coefficients of variation varied by >20%, samples were re-tested.

S2.2 Healthy volunteer's cohort

The objective of this study was to determine the levels of four specific thioethers in the breath of healthy individuals over a period of three consecutive days. We compared these results with those found in CHMI trials.

The entire study was approved by the CSIRO Health and Medical Research, Human Research Ethics Committee (proposal number: LR 4/2017). Written informed consent was received from participants prior to inclusion in the study.

The primary outcome was to measure levels in counts of allyl methyl sulphide, 1-methylthio-propane, (*E*)-1-methylthio-1-propene, (*Z*)-1-methylthio-1-propene and the ratio: (Z/E) 1-methylthio-1-propene. Inclusion criteria were: CSIRO staff based at Black Mountain laboratories in Canberra Australia, non-smoking adults aged between 18 and 45 years and must be non-smokers and in good health. Exclusion criteria were: presence of current or suspected serious chronic diseases such as cardiac or autoimmune disease (HIV or other immune deficiencies) and recent acute infectious disease or fever (e.g., sub-lingual temperature ≥ 38.5°C) within the two days prior to the start of breath collection).

There were eight participants. Each participant provided: four breath samples containing at least 1 liter of breath each day at the following times, in the morning before breakfast (around 7:00), 11:00, 15:00 and 19:00. Samples were collected from all participants for three consecutive days. The 7:00 and 19:00 samples were collected by the volunteers in their

homes. A total of 96 samples was collected. Breath samples were collected as described in Methods section in the main manuscript.

S2.3 Ambient air collection, breath transfer and analysis

Ambient air sample collection. For *P. vivax,* every time a breath sample set (N= 8) was collected was collected, we collected three ambient air samples. For healthy control study, we followed the same procedure as per *P. vivax*.

For *P. vivax* CHMI trial, 1 litre of ambient air was collected in a 3-L Sample Pro FlexFilm bag (SKC Inc, Pennsylvania), the ambient air was then transferred to a sorbent tube using an electric pump as described below. For the healthy control trials, 1 L of ambient air was collected directly into sorbent tubes using an electric pump (flow: at 200 mL min^{-1}).

Transfer of sample into sorbent tubes and storage. Breath and ambient air from the bags were transferred to sorbent tubes using an electric pump. 1 L of the sample at 200 mL min^{-1} from the bag to the sorbent tube, so all tubes had consistently the volume of sample. The tubes had two layer sorbents comprising 200 mg of Tenax TA and 200 mg of Unicarb (Markes International Limited, UK). After capture of breath volatiles onto sorbent tube they were kept at 4°C for storage and transport.

For the *P. falciparum* cohort, breath samples were stored between 7-29 days. For the *P. vivax* cohort, breath samples were stored between 7-26 days and ambient air samples were stored between 23-71 days. For healthy controls, breath samples and ambient air samples were stored between 1-12 days.

Original concentration of the thioethers on the day of collection, were calculated by correcting for decay during storage time using the method described in Section S2.4.

Standards run with breath samples. For analysis of breath collected, a mixture of external standards was analyzed with the GC-QTOF MS alongside the breath samples to correct for changes in instrument sensitivity over time. We analyzed one external standard at the start of the run and one external standard at the end of each batch of breath samples. The standard used was EPA 8240B Calibration Mix (2-Butanone, Isobutanol, 4-methyl-2-pentanone and 2-hexanone). One mL 2000 μg mL^{-1} vial standards, were purchased from Sigma-Aldrich (Australia). To spike the mixture into a sorbent tube, a 20 μg mL^{-1} solution was prepared in HPLC grade methanol. Using a solution loading rig (Markes International Limited, UK), 1μL of the solution was spiked into a sorbent tube, Tenax TA 35/60 and Sulficarb 40/70 (Markes International Limited, UK). The sorbent tube was flushed for 3min with nitrogen at a flow of 100 mL min^{-1} and analyzed by GC-QTOF. A solvent delay of 3.75 min was used for EPA 8240B Calibration Mix.

Breath analysis run schedule. For each day of analysis, we schedule the breath analysis in the following order: clean tube, tube containing mixture of external standards, breath samples, tube containing mixture of external standards and clean tube.

Quadruple time of flight GC-MS analysis. For GC-QTOF analysis, tubes were thermally desorbed for 15 min at 280°C (Unity2, Markes International, UK) and transferred to a cold trap (Inert Sulphur trap, Markes International, UK), held at 30°C and subsequently heated to

280°C, to minimize band broadening. The trap flow of 37 mL min^{-1}, split flow of 3.3 mL min^{-1} and column flow of 1.9 mL min-1 resulted in a split ratio of 2.8:1 after the cold trap.

A gas chromatograph (7890B Series GC, Agilent Technologies, USA) equipped with a HP-5MS UI GC capillary column (Agilent J&W GC Column) 30 m in length, 0.25mm ID and 0.25 μm film thickness was used with the following temperature program. Initial temperature was 35°C, held for 5min, ramped to 250°C at 5°C min^{-1} and final temperature of 250°C held for 2min. The total run time for the analysis was 50 min. The helium carrier gas flowed at a rate of 1.9 mL min^{-1}.

The QTOF (Agilent Technologies, USA) used an electron ionization source set at a temperature of 230°C. The quadrupole was set to a temperature of 150°C and the collision cell had a nitrogen flow of 1.5 mL min^{-1}. The emission current was fixed at 35 μA and the electron energy at 70eV. The mass range was scanned from 35 to 350 amu at an acquisition rate of 5 spectra sec^{-1} and a scan time of 200 ms spectrum^{-1}. Deconvolution was used to identify the compounds while ion extraction was used to calculate the area under peak for each thioether. The spectra was analyzed using Mass Hunter Qualitative analysis, Version B.07.00. In addition, chemical standards were used to confirm the identities of the compounds.

The peak areas of allyl methyl sulphide, 1-methylthio propene (E) and (Z) where determined by extracting the m/z= 73 and 88. 1-Methylthio propane by determined by extracting m/z= 61 and m/z=90 (Supplementary Table S7). Fold change used in this paper was calculated as the ratio between the final peak area and the initial peak area.

Supplementary Table 7: Exact mass of thioethers and ions used for semi-quantification and further statistical analysis

Compound	Exact Mass GC-QTOF	Average RT of compound in breath sample (min)	Mass to charge ratio *(m/z)*
Allyl methyl sulphide	88.0341	3.9	73.011, 88.034
1-Methylthio propane	90.0498	4.2	61.010, 90.049
1-Methylthio propene (E)	88.0341	4.4	73.011, 88.034
1-Methylthio propene (Z)	88.0341	4.7	73.011, 88.034

S2.4 Thioether decay correction due to storage

Thioethers are relatively unstable compared to many other breath volatiles. Thioethers have low boiling points (88-90°C) and chemically reactive double bonds. CHMI breath samples had to be transported from Brisbane to Canberra (1,100 km), kept at 4°C and stored for a variable number of days prior to analysis. We performed an experiment to determine the stability of thioethers on sorbent tubes when stored at cold temperatures in order to correct estimates of thioether concentrations for decay during storage.

Briefly tubes spiked with known amounts of thioethers were stored at four different temperatures from 6-60°C. Using Arrhenius equation, we established the decay rates for each of the thioethers. The information on the stability of thioethers allowed us to estimate the original concentration of the thioethers at the time of collection by correcting for decay. Full results of the stability studies will be published elsewhere.

S2.5 Mass spectral pre-processing and feature selection for untargeted search of novel biomarkers in *P.vivax* trial.

Given that the thioethers did not show to have predictive value for *P. vivax*, we initiated an untargeted search for volatiles associated with *P. vivax* infection. We took an automated, machine learning approach. In order to identify features of the data that can distinguish breath healthy controls and *P. vivax* infected individuals and to identify volatiles that are capable of supporting such a classification, we calculated the mutual information (MI) between each "feature", which is the unique combination of a specific value of mass to charge ratio (*m/z*) and GC retention time (Rt), and the time course (i.e. stage of the infection).

This involved the following steps:

Normalization. Samples analyzed by the GC-MS-QTOF instrument on different days were normalized using a spiked mixture of standards as described in Section S2.3. Only one standard (i.e. 2-hexanone) analyzed at the beginning of the run on each day of analysis was used for normalization. The normalization was as follows:

$$x_i' = \frac{x}{y} \times 10^7$$

where x is the original sample reading, y is the relevant day's 2-hexanone standard reading and $x_i{}'$ is the normalized reading. The factor 10^7 is used to correct for compound levels.

Peak detection and area under the curve (AUC). Next, we used an automated process (6) to identify chromatographic peaks, calculate the area under each peak and thus attribute the total counts for the particular ion in the sample. This process allowed us to compare the total amount of individual ions across samples. Automated peak detection algorithm was based on (6), which identifies a peak when the signal's second derivative is negative.

The peak detection algorithm yielded a derivative spectrum with the same number of time steps as the original data, with area under the curve at the time step of each detected peak's maximum value, and zero for all other time steps.

Peak Alignment. It is an unavoidable feature of GC that the elution time of a chemical peak can shift slightly between runs even under identical running conditions due to environmental and sample variability. To compare GC-MS data automatically, chemically identical peaks must be aligned to the same time index. This was done using a combination of piecewise alignment (7) and dynamic time warping (8). Alignment accuracy was checked using the Pearson correlation between samples (7).

Feature Selection. Feature selection identifies a subset of features, $v \in \{1, 2, 3, \ldots, m\}$, where m is the total number of features, such that the resulting subset of features gives the best classification performance for the given size constraint n on the number of features in v. We selected a subset of features by maximizing the *mutual information* between the selected features $\boldsymbol{Z^v} = \{Z^{i_1}, \ldots, Z^{i_n}\}$ and class C (9):

$$I(\boldsymbol{Z}^v, C) = \sum_{\boldsymbol{Z}^v, C} p(\boldsymbol{Z}^v, C) \frac{p(C|\boldsymbol{Z}^v)}{p(C)}$$

This approach minimizes the uncertainty about the class, given the features. The challenges in evaluating the equation above, for a given size constraint, include: (i) estimating the multivalent joint and conditional density function with only a small data set; and (ii) selecting n when there is a large number of feature sets to choose from (there are $\binom{N}{n}$, where N is the total number of features combinations for each n.

We took a simple approach to these issues. To select n features, we took the ones with the highest individual mutual information for the class. This means that only N mutual information calculations are made for a single feature and avoids the combinatorial explosion of possible feature sets as n increases (10, 11). This approach is also more appropriate for the current problem of choosing the features, which are the VOC scores after peak detection and calculation of the area under the curve, because it may allow us to identify a subset of compounds that can differentiate the classes.

References

1. Mochalski P, King J, Klieber M, Unterkofler K, Hinterhuber H, Baumann M, et al. Blood and breath levels of selected volatile organic compounds in healthy volunteers. Analyst. 2013;138(7):2134-45.
2. Cheng Q, Lawrence G, Reed C, Stowers A, RanfordCartwright L, Creasey A, et al. Measurement of Plasmodium falciparum growth rates in vivo: A test of malaria vaccines. Am J Trop Med Hyg. 1997;57(4):495-500.
3. McCarthy JS, Sekuloski S, Griffin PM, Elliott S, Douglas N, Peatey C, et al. A pilot randomised trial of induced blood-stage plasmodium falciparum infections in healthy volunteers for testing efficacy of new antimalarial drugs. PLoS One. 2011;6(8).
4. Borlon C, Russell B, Sriprawat K, Suwanarusk R, Erhart A, Renia L, et al. Cryopreserved Plasmodium vivax and cord blood reticulocytes can be used for invasion and short term culture. Int J Parasitol. 2012;42(2):155-60.
5. McCarthy JS, Griffin PM, Sekuloski S, Bright AT, Rockett R, Looke D, et al. Experimentally induced blood-stage plasmodium vivax infection in healthy volunteers. J Infect Dis. 2013;208(10):1688-94.
6. Vivo-Truyols G, Torres-Lapasio JR, van Nederkassel AM, Vander Heyden Y, Massart DL. Automatic program for peak detection and deconvolution of multi-overlapped chromatographic signals part I: peak detection. Journal of Chromatography A. 2005;1096(1-2):133-45.
7. Johnson KJ, Wright BW, Jarman KH, Synovec RE. High-speed peak matching algorithm for retention time alignment of gas chromatographic data for chemometric analysis. Journal of Chromatography A. 2003;996(1-2):141-55.
8. Clifford D, Stone G. Variable Penalty Dynamic Time Warping Code for Aligning Mass Spectrometry Chromatograms in R. J Stat Softw. 2012;47(8):1-17.
9. Battiti R. Using Mutual Information for Selecting Features in Supervised Neural-Net Learning. IEEE Trans Neural Netw. 1994;5(4):537-50.
10. Nowotny T, Berna AZ, Binions R, Trowell S. Optimal feature selection for classifying a large set of chemicals using metal oxide sensors. Sensors and Actuators B: Chemical. 2013;187(0):471-80.
11. Wang XR, Lizier JT, Nowotny T, Berna AZ, Prokopenko M, Trowell SC. Feature selection for chemical sensor arrays using mutual information. PLoS One. 2014;9(3):e89840.
